# Integrin β1 activation induces an anti-melanoma host response

**DOI:** 10.1371/journal.pone.0175300

**Published:** 2017-04-27

**Authors:** Laila Ritsma, Ipsita Dey-Guha, Nilesh Talele, Xavier Sole, Salony, Joeeta Chowdhury, Kenneth N. Ross, Sridhar Ramaswamy

**Affiliations:** 1 Cancer Center, Massachusetts General Hospital, Boston, MA, United States of America; 2 Harvard Medical School, Boston, MA, United States of America; 3 Broad Institute of Harvard & MIT, Cambridge, MA, United States of America; 4 Harvard Stem Cell Institute, Cambridge, MA, United States of America; 5 Harvard-Ludwig Center for Cancer Research, Cambridge, MA, United States of America; Duke University School of Medicine, UNITED STATES

## Abstract

TGF-β is a cytokine thought to function as a tumor promoter in advanced malignancies. In this setting, TGF-β increases cancer cell proliferation, survival, and migration, and orchestrates complex, pro-tumorigenic changes in the tumor microenvironment. Here, we find that in melanoma, integrin β1-mediated TGF-β activation may also produce tumor suppression via an altered host response. In the A375 human melanoma cell *nu/nu* xenograft model, we demonstrate that cell surface integrin β1-activation increases TGF-β activity, resulting in stromal activation, neo-angiogenesis and, unexpectedly for this nude mouse model, increase in the number of intra-tumoral CD8^+^ T lymphocytes within the tumor microenvironment. This is associated with attenuation of tumor growth and long-term survival benefit. Correspondingly, in human melanomas, TGF-β1 correlates with integrin β1/TGF-β1 activation and the expression of markers for vasculature and stromal activation. Surprisingly, this integrin β1/TGF-β1 transcriptional footprint also correlates with the expression of markers for tumor-infiltrating lymphocytes, multiple immune checkpoints and regulatory pathways, and, importantly, better long-term survival of patients. These correlations are unique to melanoma, in that we do not observe similar associations between β1 integrin/TGF-β1 activation and better long-term survival in other human tumor types. These results suggest that activation of TGF-β1 in melanoma may be associated with the generation of an anti-tumor host response that warrants further study.

## Introduction

High-grade melanoma is one of the most aggressive human tumors; it is highly metastatic and generally resistant to systemic chemotherapy [1]. For advanced melanoma patients with BRAF or NRAS mutations (28%), additional targeted drugs, while useful for controlling disease, are rarely curative due to therapy resistance [2–6]. While some advanced melanoma patients derive long-term benefit from immune checkpoint inhibitors, which have been shown to produce long-term remission, immunotherapy only cures a small minority of patients [7]. Newer insight into the nature of anti-melanoma responses might prove useful in suggesting new avenues for both fundamental and clinical investigation.

TGF-β is a cytokine that mediates a wide variety of effects within tumors [8]. In normal and premalignant cells, TGF-β leads to cytostasis and apoptosis [8]. In malignant cells with oncogenic and tumor suppressor gene mutations, however, TGF-β functions as a tumor promoter both by increasing cancer cell proliferation, survival, and migration and by inducing complex, pro-tumorigenic changes within the tumor microenvironment (TME) [8,9]. For example, TGF-β activates cancer associated fibroblasts (CAFs) and the synthesis of extracellular matrix proteins, and contributes potently to angiogenesis [9]. TGF-β also increases immune cell trafficking and differentiation–for example, it increases monocyte chemotaxis and pro-tumorigenic M2 macrophage polarization [9]. In addition, TGF-β has complex, immunosuppressive effects on T cells. While suppressing proliferation and inducing apoptosis of CD4^+^ and CD8^+^ T cells [10], TGF-β also induces T regulatory cells that can inhibit the function of both CD4^+^ and CD8^+^ T lymphocytes [10]. More recent data suggests that TGF-β may also promote T cell immunity, however, both by supporting T cell development and lineage commitment and by inducing the differentiation of T effector into T memory cells [11–14]. Given this plethora of effects, TGF-β has been explored as a target for anti-cancer therapy [15]. However, pre-clinical melanoma models have yielded conflicting results regarding TGF-β as a pro-versus anti-tumorigenic factor [16–19]. Moreover, in an early clinical trial for metastatic melanoma patients, fresolimumab, an anti-TGF-β monoclonal antibody, resulted in mixed responses without clear overall benefit [20]. Thus, the role for TGF-β as a tumor promoter or suppressor in melanoma remains unclear.

TGF-β normally resides in the TME in an inactive (latent) form [21]. Once activated, it can result in autocrine and paracrine signaling. To be activated, TGF-β must be released from the large latency complex (LLC), and bind to its cognate receptor on target cells, resulting in formation of a SMAD2/3/4 complex, which translocates to the nucleus to regulate transcription [21]. A variety of mechanisms are known to release TGF-β from the LLC, including mechanical force, proteolytic cleavage, and integrin activation [21]. Integrins are trans-membrane proteins that mediate cell communication with the microenvironment, thereby controlling cell proliferation, survival, migration, differentiation and dormancy [22,23]. Integrins mediate these processes via outside-in signaling through the activation of important signaling networks, including the PI3K-AKT and RAS-MAPK pathways, and via inside-out signaling by binding of extracellular matrix proteins or activation of growth factors like TGF-β [22]. Most widely known for conversion of latent-to-active TGF-β are integrins αvβ6, αvβ5 and αvβ8 [24–26]. In addition, integrin β1 is also known to bind the LLC, and it was recently shown that blocking of β1 integrin decreased active TGF-β levels *in vitro* [24,27], suggesting a potential role for integrin β1 in the conversion of latent-to-active TGF-β.

Here, we show that antibody-mediated activation of integrin β1 increases TGF-β signaling in the tumor microenvironment of A375 melanoma xenograft tumors. This is associated with broad-scale changes in the TME, increase in tumor-infiltrating lymphocytes, and tumor growth attenuation. When combined with paclitaxel chemotherapy long-term cures are observed. In addition, inhibition of TGF-β signaling reduces tumor infiltrating T lymphocytes and increases tumor growth. Similar associations between TGF-β / integrin β1 signaling, TME and immune-related changes, and improved survival are observed at the RNA expression level in human melanoma but not other tumor types. Combined, this suggested that TGF-β signaling might be uniquely associated with the generation of an anti-melanoma host response.

## Methods

### Cell culture

pMSCV-CMV-NLS-mCerulean construct was used to generate the mCerulean tagged A375 cell line using standard viral infection methods. mEmerald-Integrin-Beta1-N-18 was a gift from Michael Davidson (Addgene plasmid # 54129), and was used to generate stable A375-EmGFP-ITGB1 cells. A375, A375 NLS-mCerulean, A375-EmGFP-ITGB1, B16F0, 67NR, tMLEC, SK-Mel-28 and CHO-LTBP1 cells were maintained in DMEM + Glutamax, supplemented with 10% FCS, 100 U/mL penicillin, and 100 μg/mL streptomycin and were grown in a humidified atmosphere at 37°C and 5% CO_2_. The cells were mycoplasma free and were tested every two months. A375 (ATCC CRL-1619) and B16F0 (ATCC CRL-6322) cancer cells were purchased from the American Type Culture Collection (ATCC).

### TGF-β *in vitro* co-culture assay

Experiments performed according to Annes *et al* with minor adaptations [25]. CHO-LTBP1 (matrix producing) cells were plated at 2.5 * 10^5^ cells/ml and left to produce matrix. After 6 days they were removed using PBS-EDTA [15 mM]. tMLEC TGF-β reporter cells [10 * 10^4^ cells/ml] and A375 melanoma cells [7.5 * 10^4^ cells/ml], A375-EmGFP-ITGB1 cells [7.5 * 10^4^ cells/ml] or SK-Mel-28 cells [7.5 * 10^4^ cells/ml] were co-cultured in medium containing serum for 4 hours to let the cells adhere, after which the medium was replaced with serum free medium, treatments and thrombin [0.5 units/ml]. After 16–20 hours, a luciferase assay was performed according to manufacturers instructions (Promega, luciferase Assay System, E1500). The following antibody concentrations were used: ChromPure Mouse IgG, whole molecule, polyclonal ([10 μg/ml] JacksonImmuno labs 015-000-003), α-Integrin β1 activating antibody TS2/16.2.1 (TS2/16, [10 μg/ml] ATCC clone HB243), α-Integrin β1 blocking antibody P4C10 ([10 μg/ml] EMD Millipore MAB1987), Thrombin ([0.5 units/ml] Sigma Aldrich), α-TGF-β1,2,3 antibody 1D11.16.8 (1D11, [4 μg/ml] BioXCell BE0057), mouse IgG1 monoclonal ([3.3 μg/ml] R&D systems MAB1835), α-αvβ6 (clone 10D5 [100 μg/ml] abcam, ab77906), rhTGF-β1 ([2 ng/ml] R&D systems).

### TGF-β *in vitro* supernatant assay

A375 or A375-EmGFP-ITGB1 cells were plated in triplicate in 96 wells (1.2 x 10^4^ cells per well) in full serum conditions and allowed to adhere for 24 hours. Cells were replaced with serum free medium and if indicated treated with IgG ([3.3 μg/ml] R&D systems MAB1835) or α-Integrin β1 activating antibody TS2/16.2.1 (TS2/16 ([10 μg/ml] ATCC clone HB243) for 20 hours. Supernatant was collected and tMLEC reporter cells were then incubated for 16–20 hours with the supernatant. 1D11 α-TGF-β1,2,3 antibody 1D11.16.8 ([4 μg/ml] BioXCell) was added to the supernatant of some samples as a control. After incubation a luciferase assay was performed according to manufacturers instructions (Promega, luciferase Assay System, E1500).

### Immunohistochemistry/Immunofluorescence

For immunofluorescence, cells were grown directly on collagen IV-coated coverslips (Sigma). Cells were fixed in 3.7% formalin, permeabilized using 0.1% triton X-100, and treated with 0.1% SDS. For immunohistochemistry, after harvesting, organs were fixed in 1% formaldehyde, 0.2% NaIo4, 61 mM Na_2_HPO_4_, 75 mM l-Lysine and 14 mM NaH_2_PO_4_ in H2O). After fixation, the tissues were washed and placed in 30% sucrose in 61 mM Na_2_HPO_4_ and 14 mM NaH_2_PO_4_ in H2O > 6 hours and then frozen in O.C.T. Compound (Tissue-Tek). 10 μm sections were cut on a Leica CM3050 S cryotome and then stained. All samples were blocked in 2.5% BSA + 5% Normal Goat Serum (Vector labs) and then incubated with primary antibody diluted in 0.5x blocking solution, washed, and incubated with a secondary antibody. After staining, tissues were mounted with vectashield hardset with DAPI (Vector labs, H-1500). Immunofluorescence imaging was performed on a Nikon Eclipse Ti A1R-A1 confocal microscope. The following antibody dilutions were used: CD3 (1:100, ABCAM ab5690), CD4 (1:50, Santa Cruz Biotechnology sc-13573 [Clone GK1.5]), CD8α (1:50, ABCAM ab25478 [Clone 53–6.7]), αSMA-AF488 (1:50, ABCAM ab184675 [1A4]), CD31 (1:50, ABCAM ab28364), Collagen I (1:100, ABCAM ab21286), TS2/16 (1:500), pSMAD2/3 (ser 423/425)(1:100, Santa Cruz Biotechnology, sc-11769-R), Cl. Casp3 (1:100, ABCAM ab4051), KI67 (1:200, ABCAM ab15580).

T cells, KI67^+^ T cells, Cleaved Caspase 3^+^ T cells, pSMAD2/3^+^ TME cells, CAFs and endothelial cells were scored by counting the number of positive cells from multiple fields of view around the tumor perimeter at 20x magnification and calculating the average; Type I collagen fibers were scored by calculating the mean fluorescence intensity in multiple fields of view at 20x magnification and calculating the average.

### Xenograft studies *in vivo*

Animal experiments were carried out under a Massachusetts General Hospital Institutional Review Board–approved protocol (2012N000033). Animals were monitored at least once a week by a researcher, and every day by the animal caretakers. Animals were housed in groups in individually ventilated cages, with standard day/light cycles and food available *at libitum*. Tumor measurements were performed in flow cabinets under isoflurane anesthesia. Animals were euthanized by a gas mixture of O_2_/CO_2_.

For TS2/16 treatment studies *in vivo*, we injected 5×10^5^ A375 NLS-mCerulean cells subcutaneously into the flanks of 6–7 week old, female nude (*nu/nu*, Charles River Laboratories) mice. Once the tumors were palpable, mice were injected i.p. with TS2/16 (ATCC clone HB243) at 100μl at 4 mg/mL/wk, ChromPure mouse IgG (H+L) (Jackson ImmunoResearch 015-000-003) at 71.4 μl at 5.6 mg/mL/wk, or paclitaxel (Sigma T7191-5mg) at 20 mg/kg/week, for 3 weeks (long-term treatment) or 1 week (short-term treatment). Mice were sacrificed at day 28 after tumor cell injection and 4 days after the last treatment (long-term treatment), or 2 days after single treatment (short-term treatment).

For 1D11 A375 xenograft studies *in vivo*, we injected 5×10^5^ A375 NLS-mCerulean cells subcutaneously into the flanks of 6–7 week old, female nude mice (*nu/nu*, Charles River Laboratories). Once the tumors were palpable, mice were injected i.p. with 1D11 at 10.9 μl at 9.16 mg/ml/wk x 3 weeks (BioXCell, BE0057-AO25mg) or ChromPure mouse IgG (H+L) at 17.9 μl at 5.6 mg/mL/wk x 3 weeks. Tumor size (length (l), width (w) and hight (h)) was measured twice weekly by caliper, and mean tumor volume (v) was calculated: v = π/6*(l * w * h). Mice were sacrificed at day 28 after tumor cell injection and 4 days after the last treatment.

For paclitaxel/TS2/16 combination treatment studies *in vivo*, we injected 5×10^5^ A375 NLS-mCerulean cells subcutaneously into the flanks of 6–7 week old, female nude (*nu/nu*, Charles River Laboratories) or NSG (NOD.Cg-Prkdcscid Il2rgtm1Wjl/SzJ, Charles River Laboratories) mice. Once the tumors were palpable, mice were injected i.p. with paclitaxel (20 mg/kg/week x 3 weeks) followed by (TS2/16 at 100μl at 4 mg/mL/wk x 3 weeks or ChromPure mouse IgG (H+L) at 71.4 μl at 5.6 mg/mL/wk x 3 weeks) followed by paclitaxel (at 20 mg/kg/week x 3 weeks). Tumor size was measured weekly by caliper, and mean tumor volume was calculated: V = (W^2^xL)/2 (W = width, L = length). Mice were sacrificed when tumors reached an average of 130 cm^3^ in size (this was based on the size of a mouse which had to be sacrificed because its tumor reached the size limits allowed by our institutional guidelines).

### Flow cytometry

Cell staining for flow cytometry analysis was performed according to standard staining procedures. In short, cells were counted and plated at a concentration of 1*10^6^ cells per 96 well. Cells were incubated with primary antibody for 30 minutes on ice, which was followed by two washing steps and secondary antibody incubation on ice. After two washes cells were fixed, and then analysed on a BD Accuri C6 flow cytometer. FlowJo was used for final analyses: Mean fluorescence of measured antibody was subtracted by the mean fluorescence of a control antibody, and then normalized to A375 mean fluorescence. The following antibodies were used at a concentration of 1 μg / 10^6^ cells: Anti-integrin β1 antibody P5D2 (Abcam–total integrin β1), anti-mIgG1 antibody (BD–isotype control), Anti-integrin β1 antibody 12G10 (Sigma–active integrin β1), Anti-integrin β1 antibody TS2/16.

G0-like cells were measured as follows: Cells were stimulated with control IgG and TS2/16 antibody (10 μg/ml) for 3 days. Next, the cells were suspended at a concentration of 1 million cells/ml per condition and were fixed using pre-chilled 70% ethanol. The cells were washed thrice and stained with 5 μg/ml Hoechst-33342 (Sigma-Aldrich) for 45 minutes, and later stained with 1 μg/mL Pyronin-Y (Sigma-Aldrich) for 30 minutes. The cells were then washed three times with PBS, and were analyzed by BD FACSAria II. The experiment was done in triplicate and ~100,000 events per sample were collected.

### Proliferation/viability assay

*In vitro* proliferation and viability was performed according to manufacturer’s instructions (Invitrogen, LIVE/DEAD assay or Promega, CellTiter 96). Anti-TGF-β1,2,3 antibody 1D11 ([3.3 μg/ml] R&D systems), Chrompure mouse IgG ([3.3 μg/ml] JacksonImmuno labs), rhTGF-β1 ([2 ng/ml] R&D systems).

### Spheroid assay

A375 cells were plated on matrigel coated dishes and left to form spheroids for 1 day. Cultures were then treated with IgG or TS2/16 for 3 days, after which microscopy was performed. The number of spheroids containing sprouts versus no sprouts as a percentage of total spheroids was calculated and graphed.

### Westernblot

A375 cell cultures were serum starved (with 0.1% BSA) over night, then treated with IgG or TS2/16 for 0 or 2 hours. Cells were lysed using sample buffer and run under standard denaturing SDS-PAGE electrophoresis. We used the following primary antibodys: pERK 1/2000 (4370, cell signaling), GAPDH 1/10000 (abcam).

### Statistics

Statistical analyses for the pre-clinical model were performed in graphpad. When comparing two groups, a two-sided student’s t-test was performed. ANOVA was performed when comparing multiple groups, followed by a Bonferroni posthoc analysis. For all tests a P≤0.05 was deemed significant.

### Bio-informatics

See (S1 File) for methods regarding the bio-informatics [28,29].

## Results

### TS2/16 specifically activates human integrin β1

We first addressed a potential role for integrin β1 in mediating paracrine signaling by converting latent-to-active TGF-β in melanoma. To activate integrin β1 allosterically, we used TS2/16, a monoclonal antibody that specifically binds human (but not mouse) integrin β1 [30–32], which we confirmed with human A375 and mouse B16F0 cells both *in vivo* and *in vitro* (S1A–S1C Fig). We confirmed that TS2/16 activates integrin β1, as the total number of *active* β1 integrins on the cell surface measured by FACS was increased in A375 cells treated with TS2/16 (S1D Fig). A second melanoma cell line know to contain less β1 integrins, SK-Mel-28, showed similar results (S1D Fig)(expression atlas). In addition, two functional assays corroborated that TS2/16 activates integrin β1: 1. activation of integrin β1 is known to attenuate invasion by stabilizing adhesions [33]. Indeed, TS2/16 decreased 3D invasion *in vitro* (S1E and S1F Fig). 2. Activation of integrin β1 reduces G0-like cells [23]. Indeed, the percentage of these cells was reduced after treatment with TS2/16 (S1G Fig). We concluded that TS2/16 activated human integrin β1.

### Integrin β1 activation increases extracellular TGF-β activation *in vitro*

**Fig 1:**
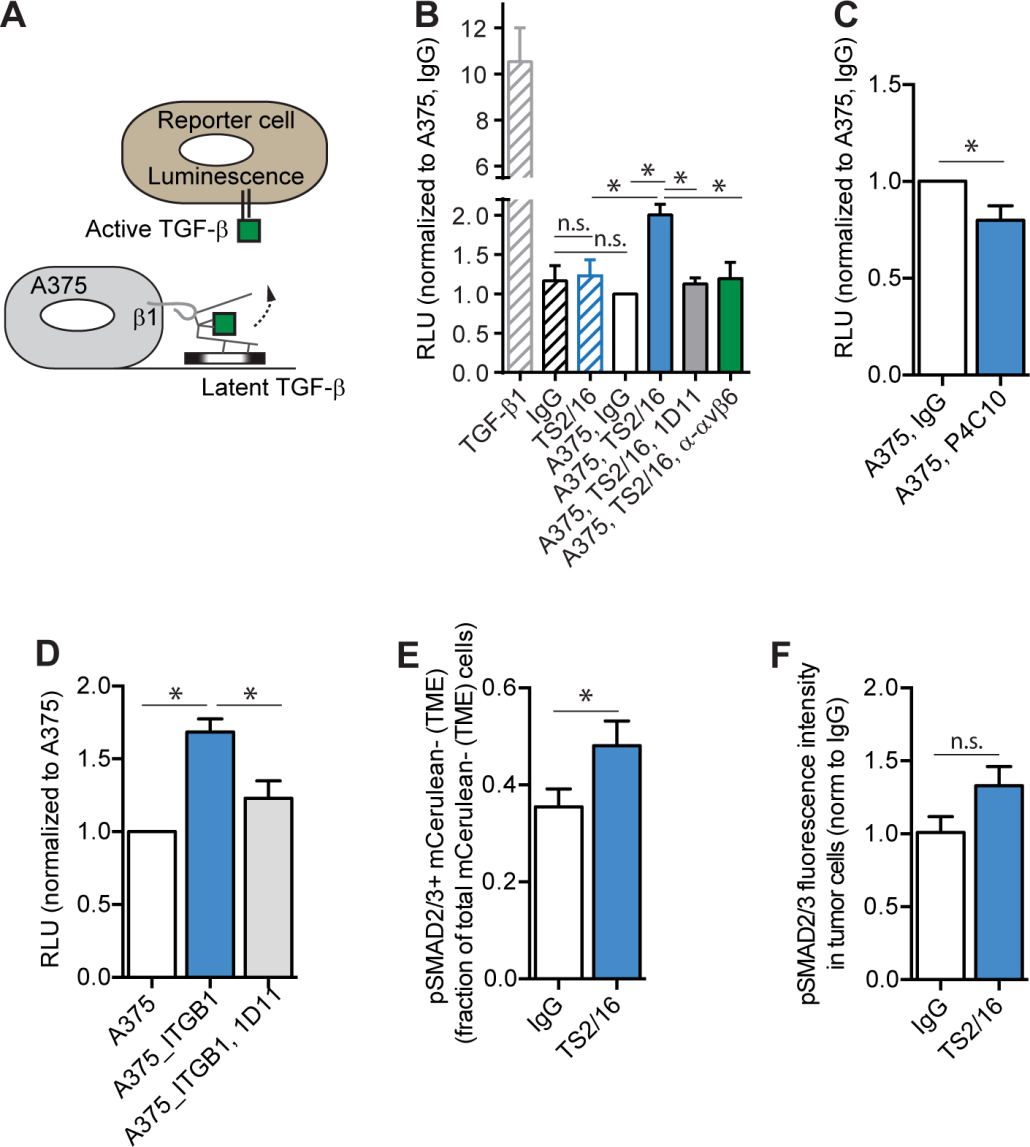
Activated integrin β1 mediates the conversion of latent-to-active TGF-β. (A) TGF-β co-culture assay cartoon explaining how TGF-β activity is measured in experiment B to D. A375 cells plated on latent-TGF-β-rich matrix are in co-culture with tMLEC reporter cells that report active TGF-β with luminescence. Integrin β1 can bind to the latent-TGF-β complex and activate TGF-β. (B—C) TGF-β co-culture assays. Quantification of the relative luciferase units (RLU—measure for active TGF-β) of tMLEC only cultures (striped bars) or tMLEC/A375 co-cultures (open bars) treated with indicated treatments for 3 days ((IgG) control antibody, TS2/16 (antibody that activates integrin β1), 1D11 (TGF-β neutralizing antibody), P4C10 (integrin β1 inhibitory antibody), α-αvβ6 (integrin αvβ6 inhibitory antibody). Graphs are normalized to A375, IgG. N ≥ 3 experiments, performed in triplicate. (D) TGF-β co-culture assay. Quantification of the RLU of tMLEC/A375 (white bar), tMLEC/A375-EmGFP-ITGB1 (blue bar), or tMLEC/A375-EmGFP-ITGB1 treated with 1D11 (grey bar) co-cultures. Graph is normalized to A375. N ≥ 3 experiments, performed in triplicate. (E) TGF-β signaling in the TME. IHC quantification on A375 NLS-mCerulean tumors treated with IgG control or TS2/16 antibody for 3 weeks. Per field of view, cells negative for mCerulean (TME cells) but with nuclear pSMAD2/3 were counted and plotted as a fraction of total mCerulean^negative^ (TME) cells. N ≥ 3 tumors per condition, 10 FOV per tumor. (F) TGF-β signaling in A375 cells. IHC quantification on A375 NLS-mCerulean tumors treated with IgG control or TS2/16 antibody for 2 days. Per field of view, pSMAD2/3 fluorescent intensity levels in mCerulean positive cells (tumor cells) was calculated. The graph is normalized to IgG control. N = 5 tumors per condition. Error bars, SEM; ANOVA (B and D) or double-sided unpaired T-Test (C, E, F): * P-value ≤ 0.05, ** P-value ≤ 0.01, n.s. P-value ≥ 0.05.

We set up a previously reported co-culture system to measure activation of TGF-β from the LLC by A375 human melanoma cells using the tMLEC mouse reporter cell line that carries a TGF-β-response element cloned from plasminogen activator inhibitor 1 (PAI-1) (Fig 1A) [25]. In this assay, relative luciferase units (RLU) are a measure for active TGF-β. We found no changes in active TGF-β when comparing tMLECs treated with IgG to a co-culture of tMLECs and A375 cells treated with IgG, suggesting the absence of a basal level of TGF-β activation by A375 cells (Fig 1B, compare IgG to A375 IgG). However, when comparing TS2/16 to IgG treatment of the tMLEC/A375 co-culture, increased TGF-β activity was observed in TS2/16 treated cultures (Fig 1B, compare A375 IgG to A375 TS2/16, S2A Fig). This increased TS2/16-induced TGF-β activity was returned to control levels when the co-culture was treated with TS2/16 and 1D11, a TGF-β neutralizing antibody (Fig 1B, compare A375 IgG to A375 TS2/16 1D11). This suggested that the increased RLU (TGF-β activity) caused by TS2/16 is indeed the result of increased levels of active TGF-β. Importantly, TS2/16 treatment of reporter cells alone did not increase the RLU compared to IgG treatment (Fig 1B, compare IgG to TS2/16). This suggested that activation of integrin β1 on A375 cells by TS2/16 increased active TGF-β. SK-Mel-28 cells, which have much lower integrin β1 levels compared to A375 cells, did not show a significant increase in active TGF-β levels (RLU) upon TS2/16 treatment in the co-culture assay (S2A Fig), confirming this hypothesis. In addition, an inhibitory antibody for integrin β1 (P4C10) reduced TGF-β activity in a tMLEC/A375 co-culture assay (Fig 1C). We also overexpressed human EmGFP-integrin-β1 in A375 cells (A375 EmGFP-ITGB1) (S2B and S2C Fig). This resulted in an increase in the total level of active integrin β1, as measured by flow cytometry using an antibody that recognizes the activated form of the protein (12G10) (S2D Fig). When A375-EmGFP-ITGB1 was compared to A375 in its ability to convert latent-to-active TGF-β, the overexpressing cell line showed a marked increase in active TGF-β (RLUs) (Fig 1D). This increase in TGF-β activity most likely resulted from an increase in active TGF-β protein, since neutralization of TGF-β with the monoclonal antibody 1D11 reduced this TGF-β activity (Fig 1D). Combined, this showed that activation of integrin β1 increases active TGF-β levels *in vitro*. This increase in TGF-β activity can be mediated by increasing the conversion of latent-to-active TGF-β and/or by increasing the secretion of active TGF-β from cells. We found no proof for the latter, as the RLUs from supernatant of A375 cells treated with TS2/16 did not differ significantly from IgG treated cells (S2E and S2F Fig). Combined, these results suggested that activation of integrin β1 increases the conversion of latent-to-active TGF-β.

As mentioned, other integrins like αvβ6 can also convert latent-to-active TGF-β, and we were interested in studying their role in our *in vitro* setup. Treatment of the co-culture with TS2/16 and an integrin αvβ6 neutralizing antibody reduced the active TGF-β levels back to control levels (Fig 1B, compare A375 IgG to A375 TS2/16 α-αvβ6). This suggested that activation of integrin β1 might indirectly lead to a conversion of latent-to-active TGF-β via the upregulation or activation of integrin αvβ6.

### Integrin β1 activation increases extracellular TGF-β activation *in vivo*

To determine if TS2/16 also increased TGF-β activity *in vivo*, we injected A375 NLS-mCerulean cells into Crl:Nu-*Foxn1*^*nu*^ (*nu/nu*) mice. Once a palpable tumor was formed, mice were treated with TS2/16 or IgG control antibody once a week for three weeks. We then performed immunohistochemistry (IHC) on tumors harvested from these mice. To determine the amount of active TGF-β in the tumor, we used pSMAD2/3 as a surrogate measure for TGF-β signaling, and observed an increased percentage of pSMAD2/3^+^ mCerulean^-^ tumor micro-environmental, but not mCerulean^+^ A375 cancer cells, when comparing TS2/16 treatment to IgG treatment (Fig 1E and 1F). These results suggested that the increase in active TGF-β observed with TS2/16 activation of integrin β1 on the cancer cell surface *in vitro* translated into an increase in pSMAD2/3 levels within the TME *in vivo*.

The increase in pSMAD2/3 levels in the TME was most likely the result of increased active TGF-β in the TME. TGF-β activity can produce broad scale micro-environmental changes, most notably activation of CAFs, production of extracellular matrix proteins, and induction of angiogenesis [9]. We therefore quantified the amount of CAFs, endothelial cells and collagen type I fibers in tumors of mice treated for 5 weeks with either TS2/16 or IgG control antibody. Indeed, we observed an increase in CAFs, endothelial cells, and Type I Collagen fibers after TS2/16 treatment compared to control (S3A to S3C Fig). Since TS2/16 recognizes human but not mouse integrin β1, our data suggested that TS2/16 is activating integrin β1 on human A375 cancer cells to induce TME changes *in vivo*, most likely by increasing the extracellular active-TGF-β levels.

### β1-integrin / TGFB1 activity correlates with stromal activation, neo-angiogenesis, and improved survival in human melanoma

**Fig 2:**
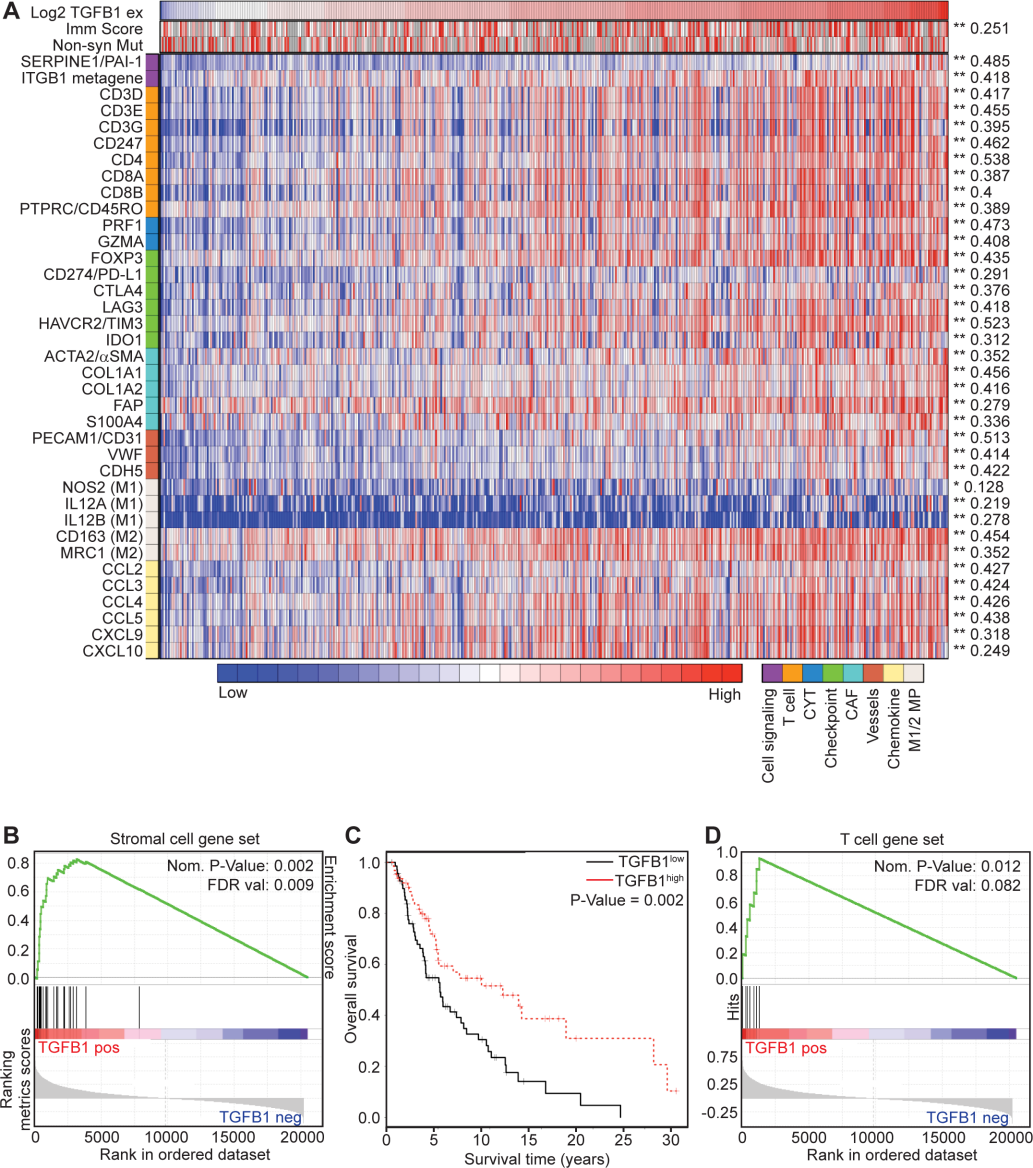
TGFB1 is positively correlated with overall survival and the TS2/16 gene signature in melanoma. (A) Heatmap showing TCGA human skin cutaneous melanoma (SKCM) patient sample Pearson correlations for TGFB1 RNA-seq expression levels and tumor microenvironmental genes. Active TGF-β signaling (SERPINE1), a metagene for integrin β1 activity (ITGB1 metagene), the immune score (imm score) and Non-Synonymous Mutation rate (non-syn Mut) are included as well. (B) Gene set enrichment analysis (GSEA) for the stromal gene signature on TCGA SKCM patient samples with genes ranked based on Pearson correlation with TGFB1 expression level. (C) Kaplan-Meier curve displaying overall survival (OS) for TCGA SKCM skin/distant melanoma patients who were subdivided into a TGFB1^high^ and a TGFB1^low^ group. TGFB1^high^ > median (N = 63), TGFB1^low^ < median (N = 70). (D) GSEA analyses for T cell gene signature on TCGA SKCM patient samples with genes ranked based on Pearson correlation with TGFB1 expression level.

We sought corroborative evidence for an integrin β1 / TGF-β1 axis mediating TME changes directly in human melanomas. Using The Cancer Genome Atlas (TCGA) dataset, we first considered all patients (i.e., primary tumors, lymph node metastases, regional skin metastases, distant metastases and other metastases) (S1 Table). Since TGF-β1 signaling increases TGFB1 expression via a positive feedback loop, we reasoned that active TGF-β1 signaling in the TME would be associated with an increase in TGFB1 transcript levels within tumors [34,35]. We first confirmed that primary and metastatic melanoma specimens express similar TGFB1 transcript levels (S4A Fig), and found a direct correlation with the expression of SERPINE1 (PAI-1), a downstream target of TGF-β signaling (which we initially used as the reporter gene in our TGF-β assay *in vitro* (Fig 1)) (Fig 2A).

We therefore considered TGFB1 / SERPINE1 expression as a surrogate measure of TGF-β activity *in vivo*. Interestingly, this TGF-β activity also correlated with expression of an experimentally validated 6-gene “metagene” (VAV2, CORO1A, EPB41L1, CCT4, FRAP1, GJB3) reflecting integrin β1 activity, further supporting a link between integrin activity and TGF-β activity in human melanoma (Fig 2A) [36]. Additional regression analyses demonstrated significant correlations between TGFB1 expression and activated stromal cells (fibroblasts (*ACTA2*, *FAP*, *S100A4*), matrix genes (*COL1A1*, *COL1A2*) and endothelial cells (*PECAM1*, *VWF*, *CDH5*), at both the single gene levels and using gene-set enrichment analysis (GSEA) (Fig 2A and 2B, S2 Table) [37,38]. Combined, these correlations corroborate the hypothesis that integrin β1 activity leads to increased TME TGF-β signaling, resulting in stromal activation and neo-angiogenesis in melanoma tumors.

CAFs, Collagens and neo-angiogenesis are strong predictors for tumor growth and worse overall survival in many types of cancer. Hence, to our surprise, patients with *TGFB1*^high^ metastases (i.e., regional cutaneous, subcutaneous (including satellite and in-transit), and distant metastases) demonstrated a statistically better overall survival compared to those with *TGFB1*^low^ metastases using Kaplan-Meier analysis (Fig 2C). We also noted a similar association between TGFB1 expression and clinical outcome when considering both primary and metastatic tumors in the TCGA dataset (S4B Fig).

Analysis of additional clinic-pathologic correlations showed that *TGFB1* expression was also positively associated with increased stage at diagnosis, despite being correlated with better survival, and also with somatic copy number alteration in a few genes (*BRAF*, *CASP8*, *TP53*, *ARID2* and *IDH1*), but not with clinically relevant point mutations (e.g., *BRAF*, *NRAS*), and (S3 Table). Given that somatic mutations are positively associated with both overall survival and response to checkpoint blockade in melanoma [39], we also asked whether *TGFB1* expression predicted for overall survival independent of mutation. However, we only observed a weak, negative correlation (-0.119) between TGFB1 and non-synonymous mutation frequency across the melanoma set (S4C Fig), and found that TGFB1 expression levels actually provided additional prognostic value beyond mutation frequency (S4D Fig).

### TGF-β positively correlates with tumor infiltrating lymphocytes in human melanoma

Our experiments in the A375 xenograft model suggested that β1-integrin / TGF-β1 activation changed the stroma within the melanoma TME, which might contribute to anti-tumor effects. However, these findings were surprising and unexpected given that CAFs, Collagen Type I fibers and blood vessels are usually associated with increased tumor growth and others have previously reported pro-tumorigenic effects of TGF-β1 in pre-clinical models of melanoma. Patient analyses corroborated the findings from our pre-clinical model, but provided no explanation as to why these TME changes, which are usually associated with tumor growth and worse overall survival, do the opposite in melanoma. Thus, we performed extensive analyses of the TME in the TCGA database that could provide us with a possible mechanism. Regression analyses demonstrated significant correlations between TGFB1 expression and: 1) the histopathologically-determined “Immunoscore” (a direct measure of T cell infiltration within tumors) and 2) transcriptional markers of T cells (e.g., *CD3*, *CD4*, *CD8*, *PTPRC*), cytolytic activity (CYT) (*PRFN1*, *GZMA*), immunosuppressive mechanisms (*FOXP3*, *CD274*, *CTLA4*, *LAG3*, *HAVCR2*, *IDO1*), macrophages (M1 (*NOS2*, *IL12A*, *IL12B*) and M2 (*CD163*, MRC1)), and chemokines (*CCL2*, *CCL3*, *CCL4*, *CCL5*, *CXCL9*, *CXCL10*), at both the single gene levels and using gene-set enrichment analysis (Fig 2A and 2D, S2 Table).

Importantly, we observed similar correlations even after removing lymph node metastases from these analyses, suggesting that these associations did not simply result from contaminating immune cells in mixed tissues (S5 Fig). These results suggested that TGF-β is not only positively associated with CAFs, collagen Type I and blood vessels, but also with immune components, including T cells.

**Fig 3:**
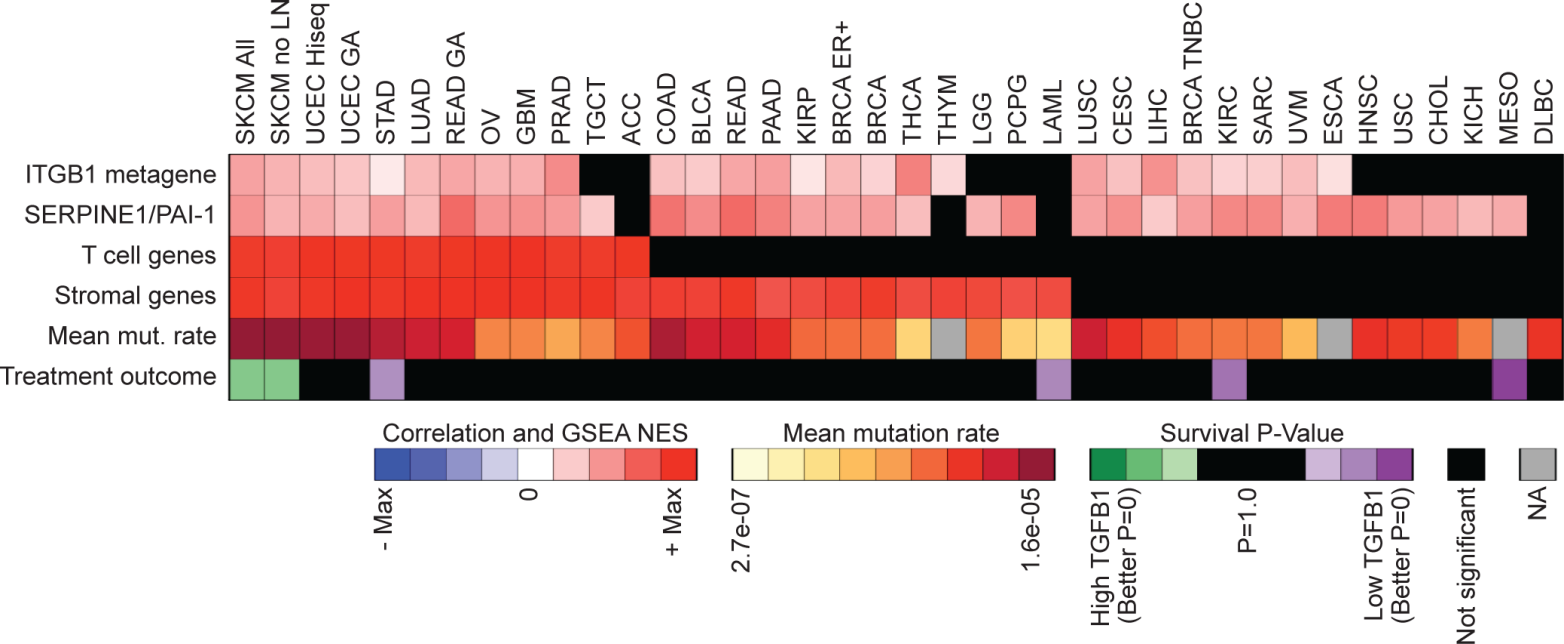
TGFB1 correlations for various tumor types. Heatmap displaying correlations between TGFB1 expression and active ITGB1 (ITGB1 metagene) and TGF-β activity (SERPINE1/PAI1) for various tumor types (S4 Table). The heatmap also includes rows with mean mutation rates (mean mut. rate), TGFB1 Kaplan-Meier treatment outcome log-rank p-values (Treatment outcome), and GSEA Normalized Enrichment Score (NES) for T-cell (T cell genes) and stromal (Stromal genes) gene sets for each of the tumor types.

To understand if these associations are melanoma specific, we examined correlations between TGF-β1 activity, integrin activation, T cell signature, stromal signature, mutation rate, and overall survival across all other tumor types within the TCGA database. Interestingly, only a subset of these different tumor types showed significant associations between *TGFB1* expression and stromal genes, and only a further subset of these showed associations with the T cell signature or, in turn, mutational load (Fig 3) (S4 Table). Malignant melanoma was the only tumor type where *TGFB1* expression significantly correlated with all four variables and overall survival, suggesting that the integrin β1 / TGF-β1 / host response phenotype we identified might be uniquely associated with anti-melanoma activity (Fig 3).

### Integrin β1 activation increases CD8^+^ T lymphocyte infiltration in A375 tumors

Next, we sought to confirm the unexpected associations we observed in the human expression data. T cells, in particular CD8^+^ T cells, are known for their anti-tumorigenic capacity and their ability to result in long-term cures. Given the positive correlation between TGFB1 and T cell genes in human melanomas, we hypothesized T cells might be responsible for the beneficial overall survival observed in TGFB1^high^ patients. *Nu/nu* mice lack a functional thymus, which is associated with a dramatic reduction in T lymphocyte counts, thus enabling successful xenografting of human cancer cells. Nevertheless, these mice continue to produce small numbers of functional T lymphocytes through extra-thymic development [40]. To test the effect of TS2/16 and TGF-β on T cells in a preclinical model, we considered using an isogenic or genetically-engineered mouse model of malignant melanoma. However, given the fact that the TS2/16 antibody does not recognize mouse integrin β1, and our previous experiments were performed in the A375 model, we continued performing additional correlative and mechanistic studies in the A375 *nu/nu* pre-clinical model.

**Fig 4:**
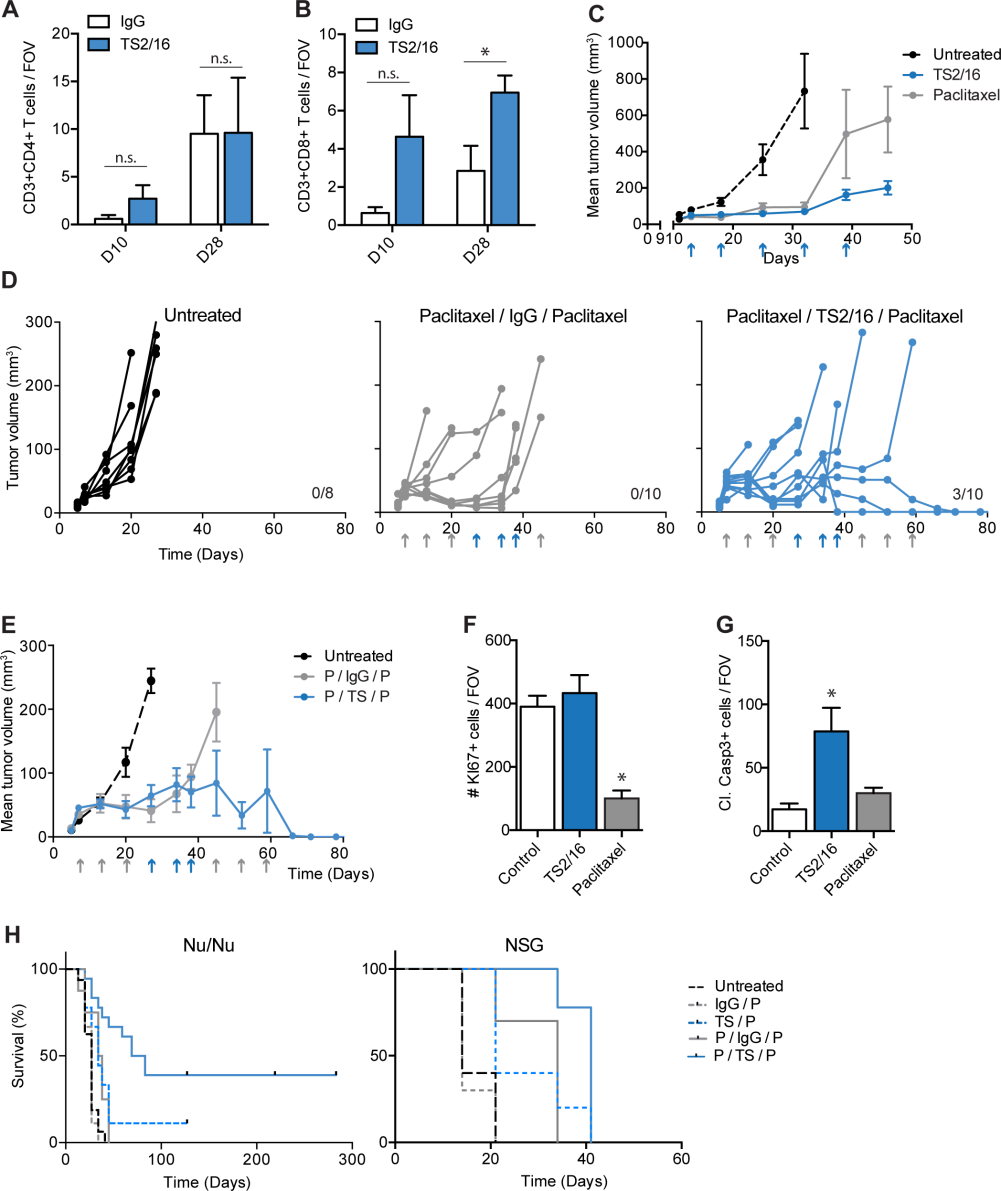
TS2/16 attenuates tumor growth by indirectly increasing apoptosis. (A-B) IHC quantification on A375 NLS-mCerulean tumors treated with IgG control (white bars) or TS2/16 (blue bars) antibody. The number of (A) CD3^+^CD4^+^, (B) CD3^+^CD8^+^ T cells was counted per field of view (FOV), and the average of ≥5 FOV in ≥3 tumors per condition was plotted in bar graphs. D10: tumors were treated once and were harvested 10 days after injection (2 days after treatment). D28: Tumors were treated 3x and were harvested 28 days after injection (5 days after last treatment). (C) Mean tumor volume of A375 tumors in *nu/nu* mice left untreated (dashed line), treated with TS2/16 (blue line) or Paclitaxel (grey line). Treatments are indicated with an arrow. N ≥ 5 mice per group. (D) A375 tumor growth measurements in *nu/nu* mice. Each curve represents the growth of a single tumor. Treatments are indicated with an arrow: TS2/16 or IgG (blue), paclitaxel (grey). Mice were sacrificed when tumors reached > 130 mm^3^, cured mice are indicated by the number on the right. N ≥ 8 tumors per group. (E) Mean tumor volume of A375 tumors shown in D. Mice were left untreated (dashed line), treated with paclitaxel/IgG/Paclitaxel (grey line) or treated with paclitaxel/TS2/16/paclitaxel (blue line). Treatments are indicated with an arrow: TS2/16 or IgG (blue), paclitaxel (grey). Mice were sacrificed when tumors reached > 130 mm^3^. N ≥ 8 mice per group. (F-G) IHC quantification on A375 NLS-mCerulean tumors treated with IgG control (white bars) or TS2/16 (blue bars) antibody for 5 weeks. The number of KI67^+^ cells (proliferation, F) or Cl. Casp3^+^ cells (apoptosis,G) per FOV was measured. N ≥ 10 FOV per condition. (H) Kaplan Meier survival curves of *nu/nu* and NSG mice respectively injected with A375 tumor cells and treated with indicated treatment regimen: IgG (IgG control antibody), P (paclitaxel), TS (TS2/16). Mice were sacrificed when tumors reached >130 mm^3^. N ≥ 10 mice per group. Survival analyses with Bonferroni post-hoc: in both *nu/nu* and NSG mice IgG/P vs TS/P and P/IgG/P vs P/TS/P was significant (P-value≤ 0.05). Error bars, SEM; Two-sided unpaired T-tests (A—B) or 1-way ANOVA with Bonferroni post-hoc test comparing treatments to control (F—G): * P-value ≤ 0.05, n.s. P-value > 0.05.

We performed IHC for CD3^+^CD4^+^ T cells and CD3^+^CD8^+^ T cells in TS2/16-treated and control tumors grown in *nu/nu* mice. As expected, only a small amount of T cells was observed in control treated mice (Fig 4A and 4B, S6A Fig). Despite the comprised T cell compartment in these mice, we noted an increase in CD3^+^CD8^+^ T cells within TS2/16-treated tumors, whereas CD3^+^CD4^+^ T cells showed no significant difference after TS2/16 treatment (Fig 4A and 4B, S6A Fig). Thus, we could confirm increased levels of tumor infiltrating CD8^+^ T lymphocytes in our preclinical model, despite making use of an immune compromised mouse model.

### Integrin β1 activation attenuates A375 tumor growth

We wondered if TS2/16 treatment would, as predicted by the human data, result in improved overall survival in our model. Overall survival is inversely correlated to tumor growth, and recently Schwartz et al observed that activation of integrin β1 by TS2/16 treatment in M21 human melanoma xenografts resulted in tumor growth attenuation [41]. Thus, these findings are in agreement with our data. However, they did not investigate if in their model integrin β1 activation resulted in TGF-β signaling activity and TME changes. Thus, we asked if in our model the effect of TS2/16 on A375 tumor growth was similar to the effect observed with M21 tumors. As a positive control for tumor growth attenuation we treated a separate group of mice with paclitaxel, a widely used chemotherapy that has been used clinically for melanoma. We injected A375 NLS-mCerulean cells into the flanks of *nu/nu* mice, left the mice untreated or treated the mice with TS2/16 or paclitaxel chemotherapy, and performed weekly measurements of tumor growth. Indeed, TS2/16 resulted in growth attenuation, to a similar extend as paclitaxel chemotherapy (Fig 4C). Thus, activation of integrin β1 is positively associated with TGF-β signaling, profound TME changes, and tumor growth attenuation in the A375 preclinical model.

Next, we asked if integrin β1 activity was able to potentiate paclitaxel, a widely used chemotherapy that has been used clinically in melanoma, and would lead to survival benefit similar to the human data. We therefore injected nude mice with A375 mCerulean-NLS tumor cells and, when palpable tumors formed, treated these mice sequentially with paclitaxel, followed by TS2/16 followed by paclitaxel [42]. As expected, treatment with this combination resulted in better inhibition of tumor growth compared to paclitaxel chemotherapy alone (Fig 4D and 4E). In addition, 30% of combination-treated tumors completely disappeared resulting in long-term survival (Fig 4D). Combined, our preclinical data confirm the human data suggesting that TGF-β1 and integrin β1 activity are positively associated with survival.

To provide a potential mechanistic explanation as to why TS2/16 attenuates tumor growth and promotes survival, we measured the direct effect of TS2/16 on intracellular signaling. TS2/16 resulted in increased levels of integrin β1 on the cell surface (S6B Fig) and increased levels of integrin β1 in the active conformation (S1D Fig), but this did not result in increased MAPK signaling (S6C Fig). Functionally, no effect on proliferation or viability was observed when cells were treated with TS2/16, whereas paclitaxel, known to affect these processes *in vitro*, reduced both processes (S6D and S6E Fig). *In vivo* analyses of tumor cell proliferation and apoptosis were obtained by performing immunohistochemistry on A375 tumors from mice treated with TS2/16 or paclitaxel for 5 weeks for respectively KI67 and cleaved caspase 3. TS2/16 treatment did not result in significant changes for KI67 (Fig 4F), however, it did increase the number of cleaved caspase 3 positive cells (Fig 4G). This discrepancy between *in vitro* and *in vivo* results might be explained by an indirect effect of TS2/16 on apoptosis, for example via the observed TME changes. To test for this, we performed similar stains on A375 tumors from mice treated with IgG or TS2/16 for only two days. No changes in TME were observed at this time point (S6F and S6G Fig), and the number of cleaved caspase 3 positive cells was similar between control and TS2/16 treated mice (S6H Fig). This suggested that TS2/16 attenuates tumor growth by indirectly, most likely via the TME, increasing apoptosis in the tumor.

### TS2/16 effects depend partially on host immune response

As we observed a correlation between TGFB1, chemokines, and immune cells in the human TCGA dataset, we hypothesized that CD8^+^ T cells might be a candidate for attenuating tumor growth and contributing to long-term cures in the A375 model. We therefore asked if TS2/16-mediated long-term survival effects might depend on an immune component. We examined the survival of A375 tumor bearing *nu/nu* mice along five different treatment arms: 1) untreated, 2) mIgG followed by paclitaxel, 3) TS2/16 followed by paclitaxel, 4) paclitaxel followed by mIgG followed by paclitaxel, 5) paclitaxel followed by TS2/16 followed by paclitaxel. Similar to before, we observed that a combination treatment with TS2/16 and paclitaxel resulted in long-term cures (Fig 4H). Repeat of the survival experiment in severely immunosuppressed NOD scid gamma (NSG) mice, which lack a functional immune system including T cells, did not produce long-term survivors after TS2/16 / paclitaxel combination treatment (Fig 4H). In contrast, overall survival was still increased when comparing IgG / paclitaxel treatment to TS2/16 / paclitaxel treatment (Fig 4H). These results suggested that the long-term cures but not the improved overall survival we observed upon activation of integrin β1 were very possibly associated with the action of a functional immune system.

### TGF-β mediates CD8^+^ T cell infiltration in A375 xenograft tumors

**Fig 5:**
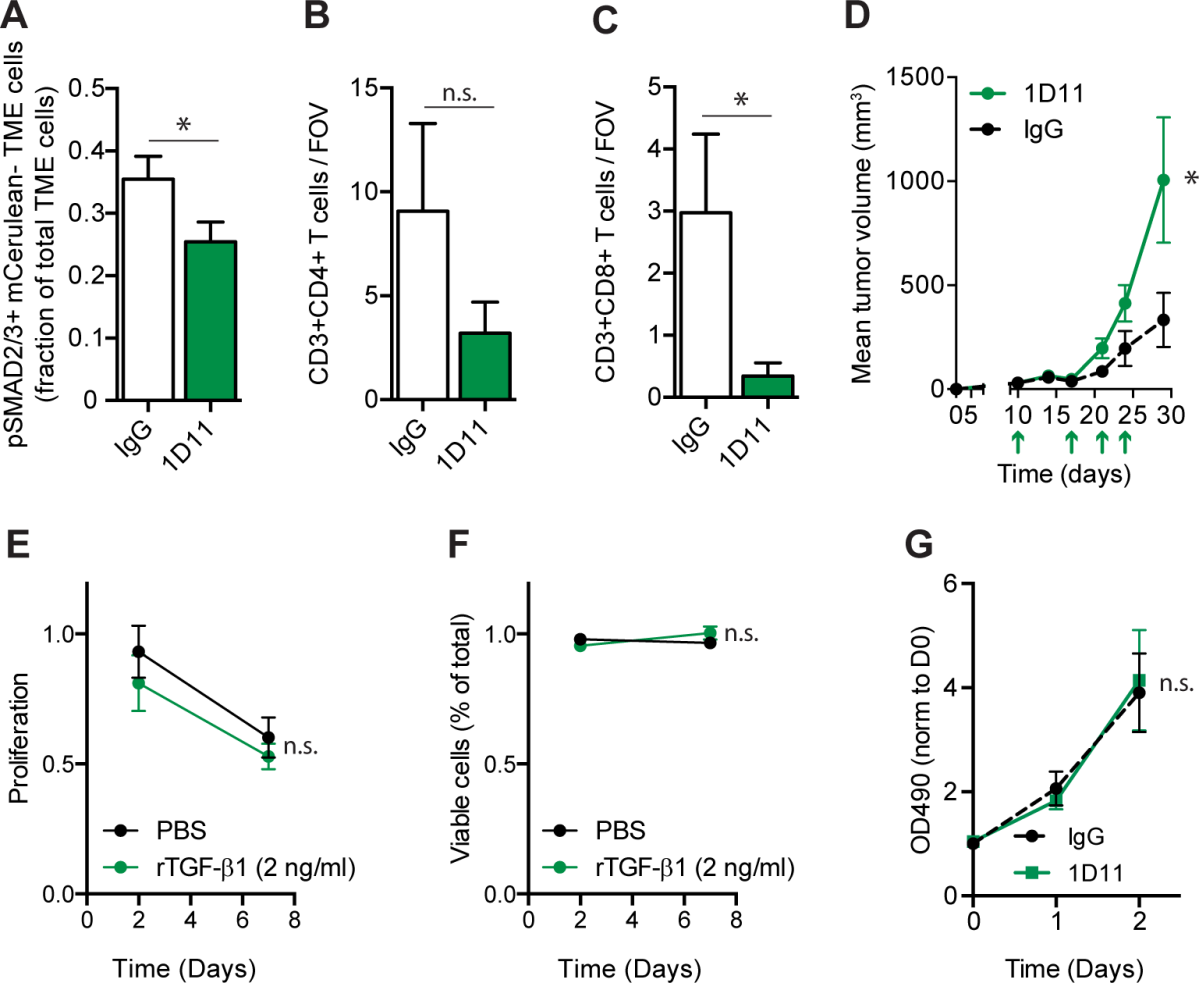
Neutralization of TGF-β reduces tumor infiltrating lymphocytes and A375 tumor growth. *(*A) TGF-β signaling in the TME. IHC quantification on A375 NLS-mCerulean tumors treated with IgG control (white bars) or 1D11 (green bars) antibody for 3 weeks. Per field of view, cells negative for mCerulean (TME cells) but with nuclear pSMAD2/3 were counted and plotted as a fraction of total mCerulean^negative^ (TME) cells. N ≥ 4 animals per condition. (B-C) IHC quantification on A375 NLS-mCerulean tumors treated with IgG control (white bars) or 1D11 (green bars) antibody. The number of (B) CD3^+^CD4^+^, (C) CD3^+^CD8^+^ T cells was counted per field of view (FOV), and the average of ≥4 FOV in ≥4 tumors per condition was plotted in the bar graphs. (D) Mean tumor volume of A375 NLS-mCerulean tumors in *nu/nu* mice treated with IgG control antibody (dashed black line) or treated with 1D11 (solid green line). Treatments are indicated with an arrow. N ≥ 6 mice per group. (E) *In vitro* proliferation assay. A375 cells were treated with PBS or rTGF-β1 for 2 or 7 days. Proliferation was measured by calculating green fluorescent (living) cells and comparing it to a live control. N = 3 experiments performed in triplicate. (F) *In vitro* cell viability assay. A375 cells were treated with PBS or rTGF-β1 for 2 or 7 days. Viability was measured by calculating the red fluorescent (dead) cells and comparing it to a dead control. N = 3 experiments performed in triplicate. G) *In vitro* proliferation assay. A375 cells were treated with IgG control antibody or 1D11 antibody for 0, 1 or 2 days. After addition of cell titel 96 aquaous solution proliferation was assessed by measuring optical density at 490 nm and normalizing it to day 0. N = 3 experiments performed in triplicate. Error bars, SEM; Double-sided unpaired T-Test (A to C) or repeated measures two-way ANOVA (D-G): * P-value ≤ 0.05, n.s. P-value > 0.05.

Our initial experiments suggested a role for TGF-β in TS2/16 treatment of tumors. As such, we were interested in dissecting the role of TGF-β in increasing tumor infiltrating CD8^+^ T lymphocytes in A375 tumors, and their role in tumor growth attenuation. We accomplished inhibition of TGF-β signaling *in vivo* using the 1D11 neutralizing antibody. As expected, pSMAD2/3 levels were reduced in the TME (Fig 5A). Moreover, 1D11 reduced CD3^+^CD8^+^ but not CD3^+^CD4^+^ T cells within A375 tumors *in vivo* (Fig 5B and 5C). To assess the effect of increased TGF-β signaling on CD8^+^ T cell proliferation or apoptosis, we performed IHC for CD8 and KI67, a proliferation marker, or cCASP3 (cleaved caspase 3), a marker for apoptosis. The percentage of KI67^+^CD8^+^ cells was not significantly different between the two treatment groups, nor did CD8^+^ cells in either group express cCASP3 (S6I and S6J Fig). These data were most consistent with TGF-β activity promoting an increase in CD8^+^ T cell numbers through enhanced influx, rather than by altering T cell proliferation or survival.

TGF-β signaling has been shown to increase cancer progression in a variety of pre-clinical models, and increases in CAFs, extracellular matrix, and neo-angiogenesis are all associated with this progression [9,43]. In contrast, TILs are generally associated with decreased tumor progression [43]. Thus, we wondered about the net effect of TGF-β depletion on A375 melanoma growth. To our surprise, depletion of TGF-β using the 1D11 blocking antibody was associated with increased tumor growth (Fig 5D). This was likely not due to a direct effect of TGF-β on the tumor cells, as we did not observe any effect of TGF-β1 or 1D11 on A375 proliferation *in vitro* (Fig 5E–5G). Thus, the net effect of TGF-β-mediated TME changes within A375 tumors appeared to be growth attenuation.

## Discussion

Our experiments using the A375 melanoma xenograft model suggest that activation of cancer cell integrin β1 mediates conversion of extracellular latent-to-active TGF-β1. We have shown this by means of an integrin β1 activating antibody TS2/16 (*in vitro* and *in vivo*), and by genetically increasing the number of active integrins on the cell surface (*in vitro*). Several integrins are known to mediate the conversion of latent-to-active TGF-β1 including integrins αvβ6, αvβ5 and αvβ8. Our results suggest that activation of integrin β1 might indirectly mediate the latent-to-active conversion of TGF-β via integrin αvβ6, for example by activating this integrin or by increasing its expression. Moreover, we cannot exclude a role for integrin αvβ5 and αvβ8 in this process, as these integrins are also being expressed in A375 cells [44].

TS2/16 activated CAFs, remodeled the extracellular matrix, and induced neo-angiogenesis within the TME. We attribute these changes to increased active TGF-β1 levels in the TME, as pSMAD2/3 levels were elevated in TME cells but not tumor cells. Note that direct measurement of active TGF-β1 levels by IHC in the tumors was not possible. We do not expect these TME changes to be the result of changes in intracellular signaling pathways downstream of integrin β1, as TS2/16 did not seem to result in major intracellular changes: 1. *In vitro* pERK levels were not altered (most likely because it was already at its top as a result of a BRAF^V600E^ mutation [45]), 2. Viability and proliferation were not affected upon TS2/16 treatment, suggesting that AKT signaling is most likely not changed. 3. According to Chen and colleagues, pAKT levels are low in A375 as a result of an active MAPK pathway caused by the BRAF^V600E^ mutation [45]. As TS2/16 does not alter the MAPK pathway, AKT signaling is most likely not affected either. However, it should be noted that activation of integrin β1 by TS2/16 reduced the total number of cellular β1 integrins, suggesting some changes in intracellular signaling are most likely occurring. Combined, these data suggest that integrin β1 (indirectly) mediates the conversion of extracellular latent-to-active TGF-β1 in A375 cells. This corroborates earlier reports that showed that inhibition or a loss of integrin β1 results in a reduction in activation of latent TGF-β in fibroblasts [24,27].

In human melanomas we find that integrin β1 / TGF-β1 activity correlates with the coordinated expression of markers associated with stromal activation, angiogenesis, similar to the *in vivo* mouse data. Combined, these data suggest the existence of an integrin β1 / TGF-β1 axis that results in activation of CAFs and induces neo-angiogenesis in melanoma tumors. Unexpectedly, this integrin β1 / TGF-β1 axis is also associated with CD8^+^ T cell infiltration, potentiation of paclitaxel chemotherapy, and long-lasting cures in an immunocompromised model system. Similarly, in human melanomas, this axis correlates with M2 macrophages, T cell infiltration, and multiple immune-regulatory pathways. Importantly, patients with *TGFB1*^high^ Stromal^high^ TIL^high^ tumors also have a significantly better overall survival independent of mutational load compared to melanoma patients with *TGFB1*^low^ tumors. This may indicate that in melanoma tumors integrin β1 /TGF-β1 activity leads to increased CD8^+^ T lymphocyte infiltration that may be responsible for improved overall survival.

There is a growing list of data on the role of TGF-β in pre-clinical models for melanoma. Our observations that TGF-β activity suppresses tumor growth contrast with those presented by Diaz-Valdes *et al*, who showed that A375 tumor growth is attenuated by inhibition of TGF-β signaling [16]. However, they used a TGF-β blocking peptide (pP144) derived from the TGF-βRIII, which may impede signaling by multiple TGF-β family ligands including TGF-β1–3, Activin-A and some BMPs [46]. Similarly, Penafuerte and colleagues reported a tumor-promoting role for TGF-β in a B16 melanoma model using the soluble TGFβIIR as a decoy [17]. The differences in tumor model (B16 is known for secreting high levels of TGF-β) and TGF-β inhibition might explain the discrepancies with our model. Mohammad *et al* investigated the use of the TIβR small molecule kinase inhibitor SD-208 (in another human melanoma xenograft model (i.e., 1205Lu)) and observed inhibition of metastasis but no effect on primary tumor growth [47]. The use of a non-specific TIβR inhibitor makes it difficult to attribute these results directly to TGF-β signaling [48]. Others specifically overexpressed TGF-β1 in human 451Lu or WM3248 cells and observed no effect on tumor growth [18]. In these experiments, however, fibroblasts were co-injected with the tumor cells, possibly confounding the effects of TGF-β1. In contrast, we use a highly specific TGF-β antibody to neutralize TGF-β signaling in the A375 melanoma model, although we cannot discriminate between TGF-β1, 2 or 3. Similar to us, Ramont *et al* very specifically activated TGF-β1 signaling, by pre-treating B16 melanoma cells with TGF-β1 followed by peri-tumoral TGF-β1 injections, and observed anti-tumorigenic effects, consistent with our results suggesting that TGF-β1 may actually suppress melanoma growth [19]. Importantly, and unlike these other pre-clinical observations, we present additional evidence that TGF-β1 activation is associated with stromal and immune cell activation and better long-term clinical outcome in patients with metastatic melanoma, further supporting an anti-tumorigenic role for TGF-β in this specific context.

Our results indicate that TGF-β-mediated stromal activation is associated with T lymphocyte influx, whereas TGF-β is a well-known for directly and indirectly suppressing T lymphocyte development and function through multiple mechanisms, including decreasing CD4^+^ and CD8^+^ T cell proliferation and survival, and the regulation of regulatory cell function [10,41]. How might TGF-β-mediated changes in the TME lead to this pro-inflammatory phenotype? The β1 integrin / TGF-β-associated program that we observe is very reminiscent of dynamic microenvironmental changes seen in the inflammatory phase of early wound healing [39]. Certainly, TGF-β is a major initiator of wound healing process that activates macrophages, fibroblasts, and endothelial cells [49]. Monocytes are one of the first immune cell types to enter an early wound, where they are activated by TGF-β to polarize towards an M2 phenotype and produce a plethora of cytokines that promote angiogenesis and, in turn, activate local fibroblasts to secrete extracellular matrix [49]. Moreover, macrophages are a major source of CCL2, CCL3, CCL4, CCL5, CXCL9 and CXCL10, which are important chemokines that induce T lymphocyte chemotaxis, and which we find in association with stromal activation in actual melanomas [49,50]. TGF-β also directly activates CAFs to secrete a variety of growth factors and cytokines that increase cancer and stromal cell proliferation, survival, and migration to promote angiogenesis, inflammation, and tumorigenesis [51]. CAFs are the major source of extracellular matrix within tumors, particularly type I collagen, which in turn enables a variety of stromal and immune cells to migrate into and within the TME, and may also contribute to the T cell infiltration we observe in melanomas [52]. Consistent with this view, it was recently shown that CAFs restrain pancreatic cancer growth by increasing an immune response [53].

Of interest is the observed decrease in CD8^+^ T cell influx in A375 xenograft tumors upon inhibition of TGF-β signaling using 1D11, whereas TS2/16 treatment increased CD8^+^ T cells in those tumors. The immunocompromised *nu/nu* mouse model has a limited number of T cells, which have developed extrathymically. The T cell receptor (TCR) repertoire in the T lymphocytes of these *nu/nu* mice is limited [54], and certain populations of T cells (like CD4+CD25+ T regulatory cells) only develop under specific conditions [55,56]. Thus, despite showing a similar correlation between TGF-β signalling and tumor infiltrating lymphocytes in athymic mice and humans, it will be important to verify our results in an isogenic mouse model with a fully competent immune system. Moreover, in this setting it remains to be determined if indeed these tumor infiltrating lymphocytes are the primary reason TGF-β attenuates melanoma tumor growth.

Curiously, TS2/16 treatment increases CD8^+^ T cell influx into A375 tumors, but sequential combination treatment with paclitaxel followed by TS2/16 is necessary to produce long-term cures in these animals. Consistent with prior observations, we speculate that taxane chemotherapy may also contribute to an inflammatory / immune anti-tumor response by directly killing tumor cells, thereby releasing tumor antigens that are necessary for T cell priming. Furthermore, taxane chemotherapy may increase antigen presenting cell function, reduce immune suppression, and enhance T and NK cells through multiple mechanisms [57]. It is of interest that only a portion of the mice receiving combination therapy were cured. One potential explanation might relate to additional immune-regulatory mechanisms at play in the mice that are not cured. For example, in our analysis of human melanomas, the correlation between TGF-β and PRF1 / GZMA expression suggests the presence of active CTLs, while increased expression of various negative immune-regulatory markers (i.e., FOXP3, PDL1, CTLA4, LAG3, TIM3, IDO1) may reflect blunting of this anti-tumor response via compensatory mechanisms to establish a dynamic equilibrium between tumor and host during the evolution of these cancers (Fig 2A) [38,58].

Combined, these observations suggest that simple manipulations to increase or decrease TGF-β in melanoma and other contexts for therapeutic effect in the clinic may prove more complex than originally anticipated [41]. Additional work will now be required to further elucidate mechanisms and validate this complex biology, including further characterization of stromal and immune cell subsets, in additional xenografts or syngeneic and genetically engineered melanoma models with intact immune systems.

## Supporting information

S1 Fig TS2/16 specifically binds human β1 integrin. (A) B16F0 and A375 tumor cells were treated with IgG or TS2/16, then fixed and stained for IgG1 alexa fluor 488 (AF 488), a secondary antibody recognizing TS2/16. Representative picture from n = 3 experiments. (B) Histogram from FACS experiment of B16F0 (upper graph) and A375 (lower graph) tumor cells treated with IgG or TS2/16, and then fixed and stained for IgG1 alexa fluor 488 recognizing TS2/16. Representative plot from n = 3. (C) Tumors harvested from A375-mCerulean-NLS tumor bearing mice treated with TS2/16 were sectioned and stained for IgG1 alexa fluor 488 which recognizes TS2/16. mCerulean present in the tumor cells is false colored as red. M = microenvironment, H = human A375 tumor cells. Representative picture from n = 3 experiments. Scale bar, 50 μm. (D) Quantification of FACS data averaging the mean fluorescence intensity for A375 or SK-Mel-28 cells stained with 12G10, and antibody recognizing the active form of integrin β1. Cells were treated with IgG or TS2/16. Data is normalized to IgG. N = 3 experiments. (E) Micrographs of A375 cell spheroids on matrigel treated with IgG or TS2/16 for 3 days. Invading cells are indicated with an arrowhead. Quantification is shown in (F). A representative picture of N = 3 experiments is shown. (F) Quantification of spheroids with invading or no invading cells, as described and shown in (E). Per field of view, spheroids with vs without sprouts were quantified and plotted as a percentage of total spheroids. N = 6 field of views. (G) Quantification of G0-like cells as a fraction of total cells of A375 cells treated with IgG or TS2/16 for 3 days. N = 3 experiments. Error bars, SEM; two-way ANOVA with Bonferroni post-hoc test making indicated comparisons (D), Two-sided unpaired T-tests (F and G): * P-value ≤ 0.05, ** ≤ 0.01.
(TIF)

S2 Fig TS2/16 activates integrin β1. (A) TGF-β co-culture assay. Quantification of the relative luciferase units (RLU—measure for active TGF-β) of tMLEC/A375 co-cultures (open bars) or tMLEC/SK-Mel-28 co-cultures treated with IgG or TS2/16. Graphs are normalized to IgG treatment. N = 3 experiments performed in triplicate. (B) Quantification of FACS data averaging the mean fluorescence intensity for A375 and A375 EmGFP-ITGB1 cells stained with P5D2, measuring total integrin β1. Graph is normalized to A375 cells. N = 3 experiments. (C) Quantification of FACS data averaging the mean fluorescence intensity for EmGFP in A375 and A375 EmGFP-ITGB1 cells. EmGFP measures the total overexpressed EmGFP-Integrin β1. Graph is normalized to A375 cells. N = 3 experiments. (D) Quantification of FACS data averaging the mean fluorescence intensity for A375 or A375 EmGFP-ITGB1 cells stained with 12G10, an anti-integrin β1 antibody recognizing the active form of the protein. Graph is normalized to A375 cells. N = 4 experiments. (E) TGF-β assay. Quantification of the relative luciferase units (RLU—measure for active TGF-β) of supernatant from A375 cells treated with IgG (white bars) or TS2/16 (blue bars) for 20 hours. Supernatants were left untreated (open bars) or were treated with a neutralizing antibody for TGF-β (1D11, striped bars). N = 3. (F) TGF-β assay. Quantification of the relative luciferase units (RLU—measure for active TGF-β) of supernatant from A375 cells (white bars) or A375 EmGFP_ITGB1 cells (blue bars). Supernatants were left untreated (open bars) or were treated with a neutralizing antibody for TGF-β (1D11, striped bars). N = 3. Error bars, SEM; * P-value ≤ 0.05, ** ≤ 0.01, n.s. P-value > 0.05.
(TIF)

S3 Fig Integrin β1 activation by TS2/16 results in typical TGF-β-associated microenvironmental changes. (A) IHC of tumors left untreated or treated with TS2/16 for 5 weeks. The number of CD31^+^ microvessels or aSMA^+^CD31^-^ CAFs per field of view (FOV) was quantified. For Type I collagen the mean fluorescence intensity per FOV for COL1A1 was calculated. N = 10 FOV in 1 tumor per condition. (B-C) Representative micrographs used for the measurements in the graph in A. Error bar, SEM; * *P*-value ≤ 0.05, ns P-value > 0.05.
(TIF)

S4 Fig TGFB1 associations and survival analyses. (A) Histogram displaying the distribution of TGFB1 expression level of TCGA SKCM samples subdivided into groups based on tumor or metastasis type. (B) Kaplan-Meier curve displaying OS for all TCGA SKCM patients who were subdivided into TGFB1^high^ and TGFB1^low^ groups. TGFB1^high^ > median (N = 222), TGFB1^low^ < median (N = 222). (C) Scatter plot displaying the non-synonymous mutation rate of TCGA SKCM patients with mutational information versus TGFB1 expression level (Pearson correlation = -0.119, p = 0.047). (D) Kaplan-Meier curve displaying OS for TCGA SKCM patients who were subdivided into the indicated groups. For TGFB1 expression level, TGFB1 high indicates > median, TGFB1 low indicates < median and for non-synonymous mutation status, Mut high indicates ≥ P = 0.2 quantile, and Mut low indicates < P = 0.2 quantile. Kaplan-Meier log-rank P-value = 0.001.
(TIF)

S5 Fig TGF-β1 expression does not correlate with tumor stage. (A) GSEA analyses for TCGA SKCM patient samples (excluding lymph node metastases) with genes ranked based on Pearson correlation with TGFB1 expression level and testing enrichment of the tumor microenvironment gene signatures (S2 Table). (B) Heatmap showing TCGA SKCM patient sample correlations (excluding lymph node metastases) for TGFB1 RNA-seq expression levels and tumor microenvironmental genes (S2 Table).
(TIF)

S6 Fig TS2/16 does not affect CD8^+^ cell proliferation or apoptosis. (A) Micrographs of IgG or TS2/16 treated tumors stained for DAPI, CD8 and CD3. CD3+CD8+ T cells are indicated by a yellow arrow. A zoom in of the boxed area is shown on the right. Scale bar, 50 μm. (B) Quantification of FACS data averaging the mean fluorescence intensity for A375 and SK-Mel-28 cells stained with P5D2, measuring total integrin β1. Graphs are normalized to IgG control. N = 3 experiments. (C) Western blot of A375 cells treated with IgG or TS2/16 for 0 or 2 hours stained for pERK and GAPDH. (D) *In vitro* A375 proliferation assay. Proliferation is measured by analyzing the green fluorescence (Calcein-AM, 530 nm) as a measure for living cells and normalizing it to a live control. N = 3 experiments performed in triplicate. (E) *In vitro* A375 cell viability assay. Viability is measured by analyzing red fluorescence (Ethidium homodimer-1, 645 nm) as a measure for dead cells and normalizing it to a dead control. N = 3 experiments performed in triplicate. (F-H) IHC of tumors treated with IgG or TS2/16 for 2 days. The number of αSMA^+^ cells (F) or Cl. Casp3^+^ cells (H) per field of view (FOV) was quantified. For Type I collagen the mean fluorescence intensity per FOV for COL1A1 was calculated (G). N ≥ 3 mice per condition. (I-J) IHC of tumors treated with IgG or TS2/16 for 3 weeks. The number of CD8^+^ KI67^+^ or CD8^+^Cleaved Caspase 3^+^ cells was calculated as a fraction of total CD8^+^ cells per field of view. N ≥ 3 tumors per condition. Error bar, SEM; Two-way ANOVA with Bonferroni posthoc test (B), one-way ANOVA with Bonferroni posthoc test (D,E), double-sided T-test (F-J). * P-value ≤ 0.05, n.s. P-value > 0.05.
(TIF)

S1 Table List of used TCGA melanoma samples. List of TCGA melanoma samples and clinical information for samples that have both RNASeqV2 RSEM expression data and clinical data.
(XLSX)

S2 Table Gene signatures. List of genes in the tumor microenvironment signature and their correlations with TGFB1 expression.
(XLSX)

S3 Table TCGA SKCM TGFB1 clinical associations. Association of clinical and pathological parameters with TGFB1 RNA-seq expression (t-test or Pearson correlation test).
(XLSX)

S4 Table Summary table of all TCGA cancer type data sets. Summary table of outcome, GSEA results, ITGB1 meta-gene correlation and SERPINE1 correlation in all TCGA cancer type data sets.
(XLSX)

S1 File Supplementary methods. This file contains supplementary methods regarding the bioinformatics analyses that were performed.
(DOCX)
