# Supplementary figures and images for: Integrin β1 activation induces an anti-melanoma host response
Source: PLoS One. 2017 Apr 27;12(4):e0175300. doi: 10.1371/journal.pone.0175300 (PMC5407755; doi:10.1371/journal.pone.0175300)

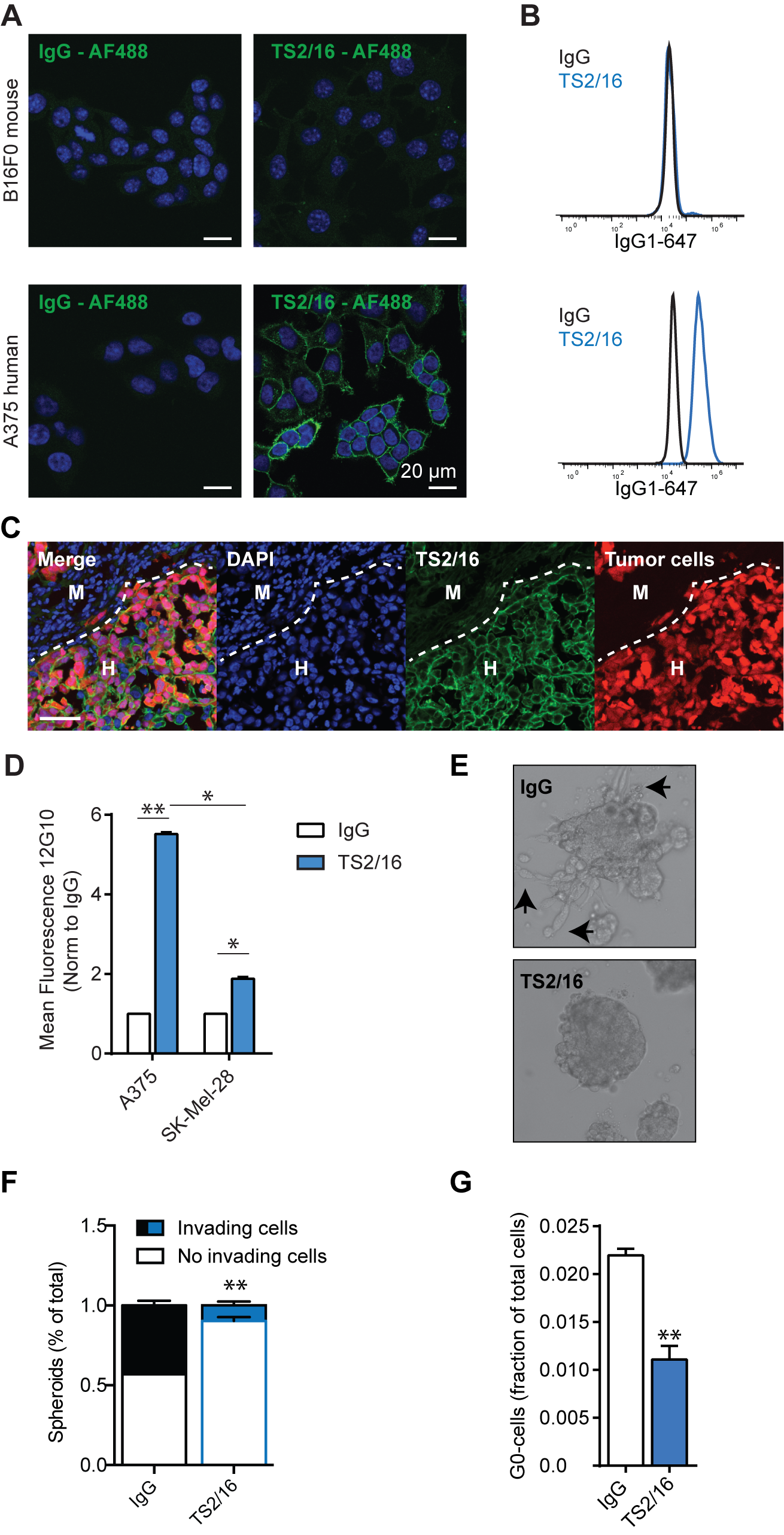

Supplement: S1 Fig — (A) B16F0 and A375 tumor cells were treated with IgG or TS2/16, then fixed and stained for IgG1 alexa fluor 488 (AF 488), a secondary antibody recognizing TS2/16. Representative picture from n = 3 experiments. (B) Histogram from FACS experiment of B16F0 (upper graph) and A375 (lower graph) tumor cells treated with IgG or TS2/16, and then fixed and stained for IgG1 alexa fluor 488 recognizing TS2/16. Representative plot from n = 3. (C) Tumors harvested from A375-mCerulean-NLS tumor bearing mice treated with TS2/16 were sectioned and stained for IgG1 alexa fluor 488 which recognizes TS2/16. mCerulean present in the tumor cells is false colored as red. M = microenvironment, H = human A375 tumor cells. Representative picture from n = 3 experiments. Scale bar, 50 μm. (D) Quantification of FACS data averaging the mean fluorescence intensity for A375 or SK-Mel-28 cells stained with 12G10, and antibody recognizing the active form of integrin β1. Cells were treated with IgG or TS2/16. Data is normalized to IgG. N = 3 experiments. (E) Micrographs of A375 cell spheroids on matrigel treated with IgG or TS2/16 for 3 days. Invading cells are indicated with an arrowhead. Quantification is shown in (F). A representative picture of N = 3 experiments is shown. (F) Quantification of spheroids with invading or no invading cells, as described and shown in (E). Per field of view, spheroids with vs without sprouts were quantified and plotted as a percentage of total spheroids. N = 6 field of views. (G) Quantification of G0-like cells as a fraction of total cells of A375 cells treated with IgG or TS2/16 for 3 days. N = 3 experiments. Error bars, SEM; two-way ANOVA with Bonferroni post-hoc test making indicated comparisons (D), Two-sided unpaired T-tests (F and G): * P-value ≤ 0.05, ** ≤ 0.01. (TIF) [file pone.0175300.s001.tif]

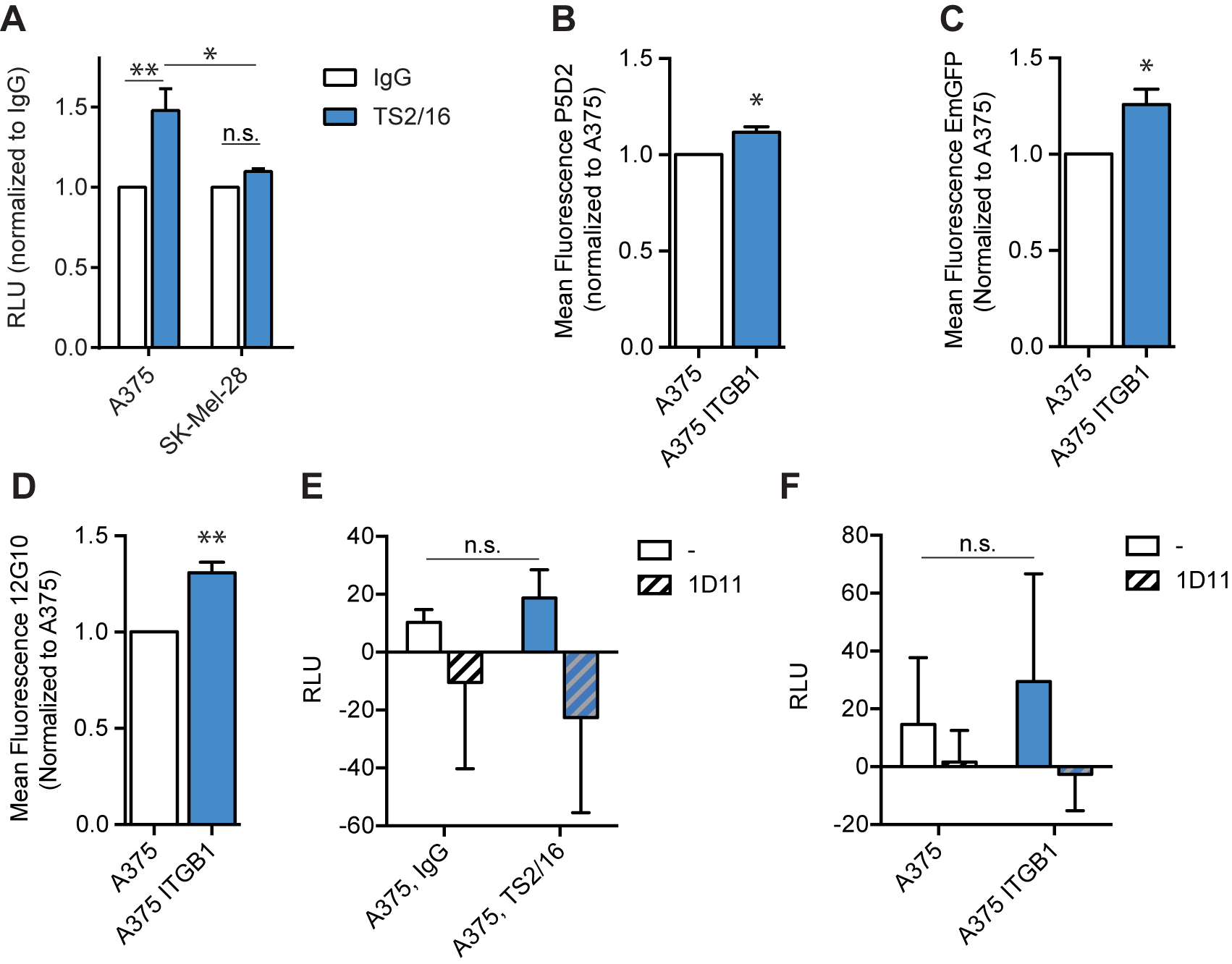

Supplement: S2 Fig — (A) TGF-β co-culture assay. Quantification of the relative luciferase units (RLU—measure for active TGF-β) of tMLEC/A375 co-cultures (open bars) or tMLEC/SK-Mel-28 co-cultures treated with IgG or TS2/16. Graphs are normalized to IgG treatment. N = 3 experiments performed in triplicate. (B) Quantification of FACS data averaging the mean fluorescence intensity for A375 and A375 EmGFP-ITGB1 cells stained with P5D2, measuring total integrin β1. Graph is normalized to A375 cells. N = 3 experiments. (C) Quantification of FACS data averaging the mean fluorescence intensity for EmGFP in A375 and A375 EmGFP-ITGB1 cells. EmGFP measures the total overexpressed EmGFP-Integrin β1. Graph is normalized to A375 cells. N = 3 experiments. (D) Quantification of FACS data averaging the mean fluorescence intensity for A375 or A375 EmGFP-ITGB1 cells stained with 12G10, an anti-integrin β1 antibody recognizing the active form of the protein. Graph is normalized to A375 cells. N = 4 experiments. (E) TGF-β assay. Quantification of the relative luciferase units (RLU—measure for active TGF-β) of supernatant from A375 cells treated with IgG (white bars) or TS2/16 (blue bars) for 20 hours. Supernatants were left untreated (open bars) or were treated with a neutralizing antibody for TGF-β (1D11, striped bars). N = 3. (F) TGF-β assay. Quantification of the relative luciferase units (RLU—measure for active TGF-β) of supernatant from A375 cells (white bars) or A375 EmGFP_ITGB1 cells (blue bars). Supernatants were left untreated (open bars) or were treated with a neutralizing antibody for TGF-β (1D11, striped bars). N = 3. Error bars, SEM; * P-value ≤ 0.05, ** ≤ 0.01, n.s. P-value > 0.05. (TIF) [file pone.0175300.s002.tif]

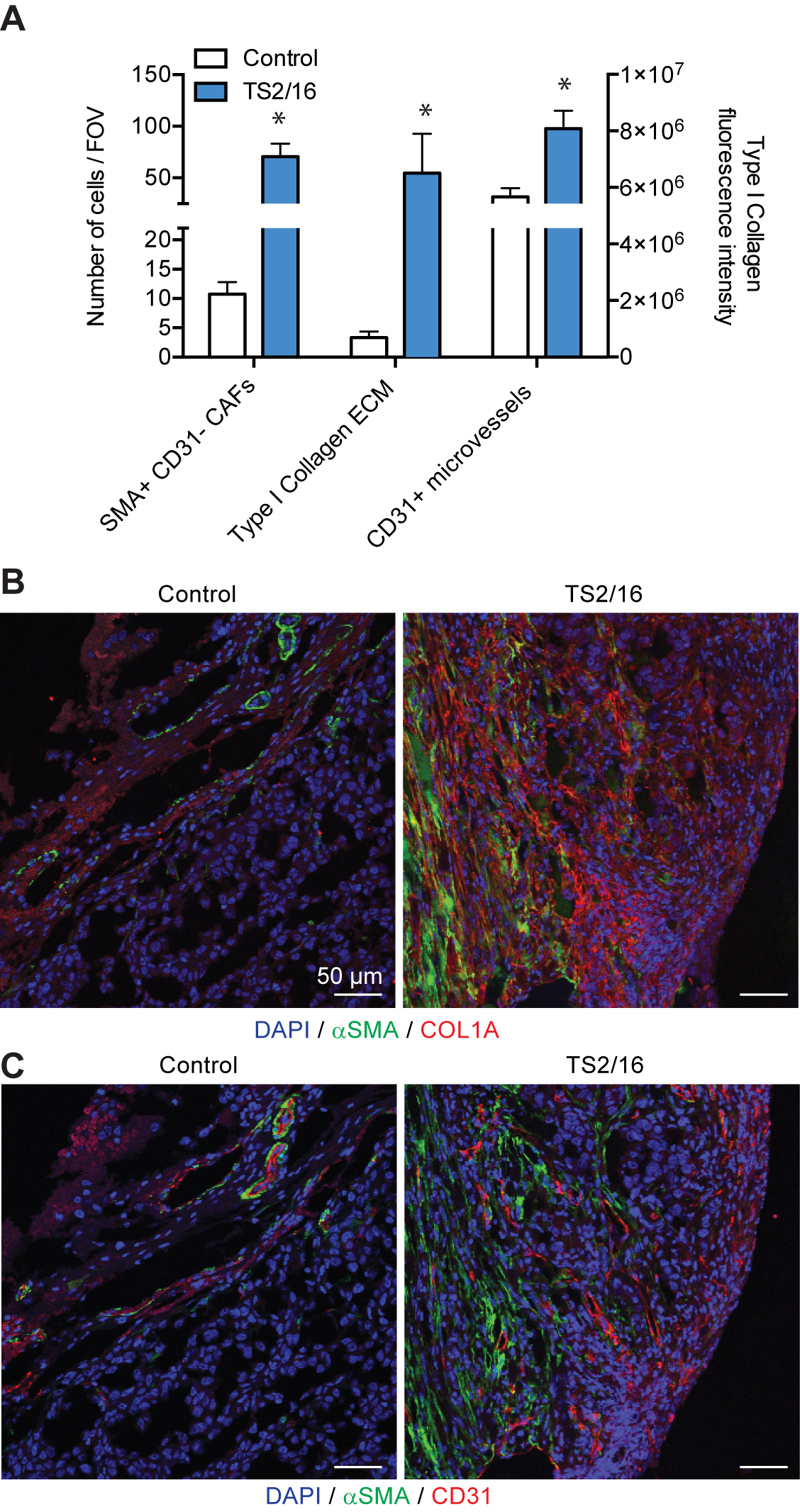

Supplement: S3 Fig — (A) IHC of tumors left untreated or treated with TS2/16 for 5 weeks. The number of CD31+ microvessels or aSMA+CD31- CAFs per field of view (FOV) was quantified. For Type I collagen the mean fluorescence intensity per FOV for COL1A1 was calculated. N = 10 FOV in 1 tumor per condition. (B-C) Representative micrographs used for the measurements in the graph in A. Error bar, SEM; * P-value ≤ 0.05, ns P-value > 0.05. (TIF) [file pone.0175300.s003.tif]

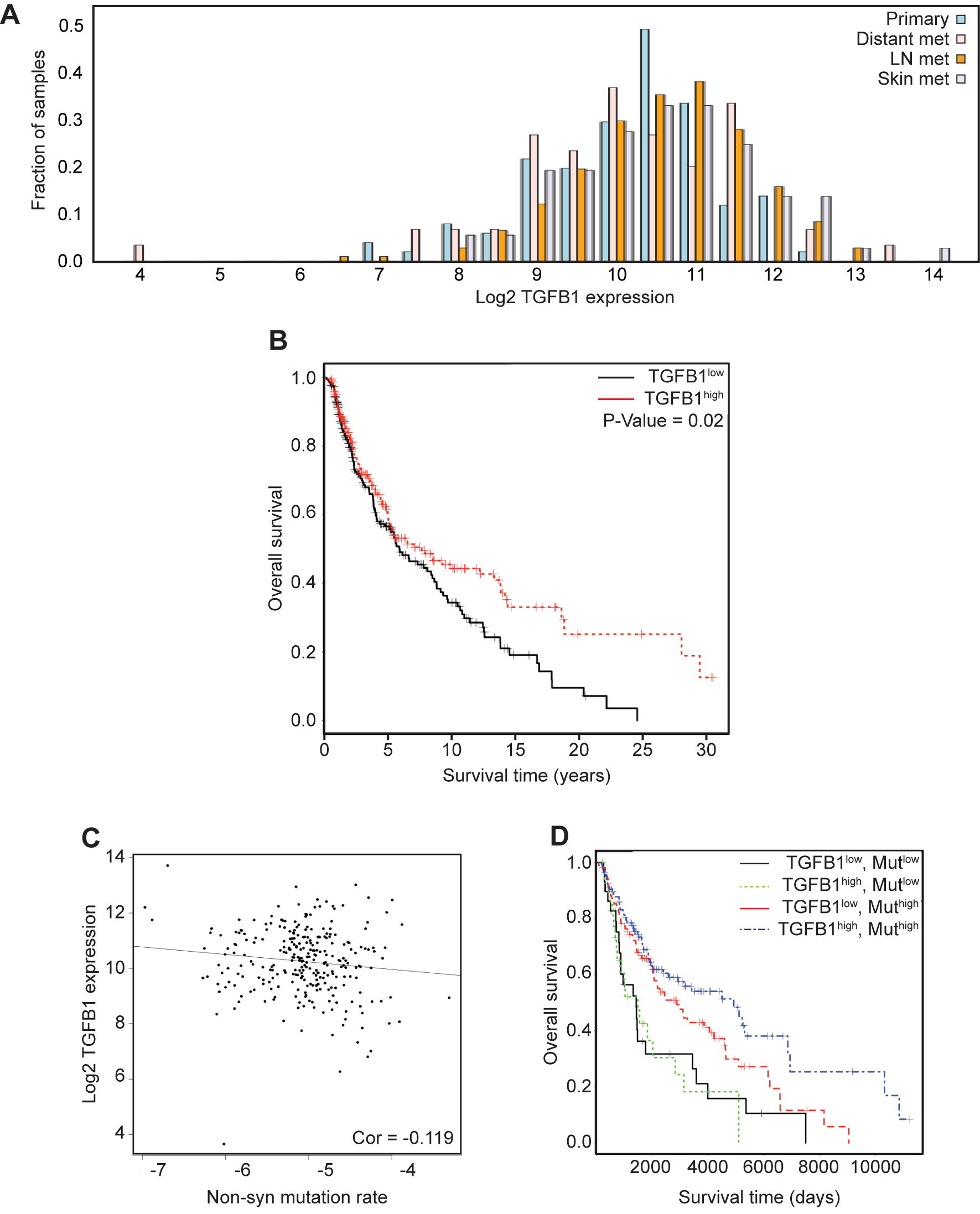

Supplement: S4 Fig — (A) Histogram displaying the distribution of TGFB1 expression level of TCGA SKCM samples subdivided into groups based on tumor or metastasis type. (B) Kaplan-Meier curve displaying OS for all TCGA SKCM patients who were subdivided into TGFB1high and TGFB1low groups. TGFB1high > median (N = 222), TGFB1low < median (N = 222). (C) Scatter plot displaying the non-synonymous mutation rate of TCGA SKCM patients with mutational information versus TGFB1 expression level (Pearson correlation = -0.119, p = 0.047). (D) Kaplan-Meier curve displaying OS for TCGA SKCM patients who were subdivided into the indicated groups. For TGFB1 expression level, TGFB1 high indicates > median, TGFB1 low indicates < median and for non-synonymous mutation status, Mut high indicates ≥ P = 0.2 quantile, and Mut low indicates < P = 0.2 quantile. Kaplan-Meier log-rank P-value = 0.001. (TIF) [file pone.0175300.s004.tif]

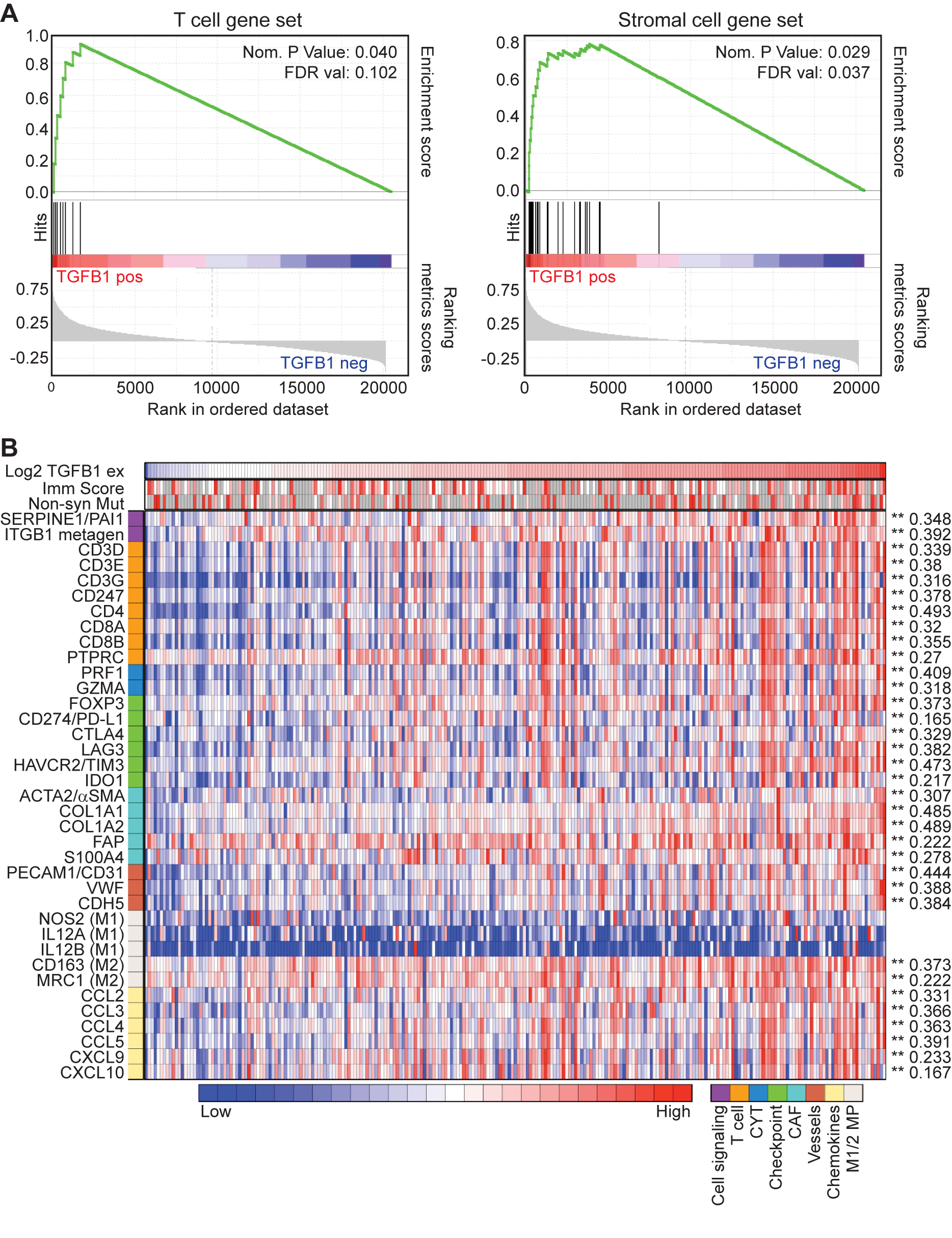

Supplement: S5 Fig — (A) GSEA analyses for TCGA SKCM patient samples (excluding lymph node metastases) with genes ranked based on Pearson correlation with TGFB1 expression level and testing enrichment of the tumor microenvironment gene signatures (S2 Table). (B) Heatmap showing TCGA SKCM patient sample correlations (excluding lymph node metastases) for TGFB1 RNA-seq expression levels and tumor microenvironmental genes (S2 Table). (TIF) [file pone.0175300.s005.tif]

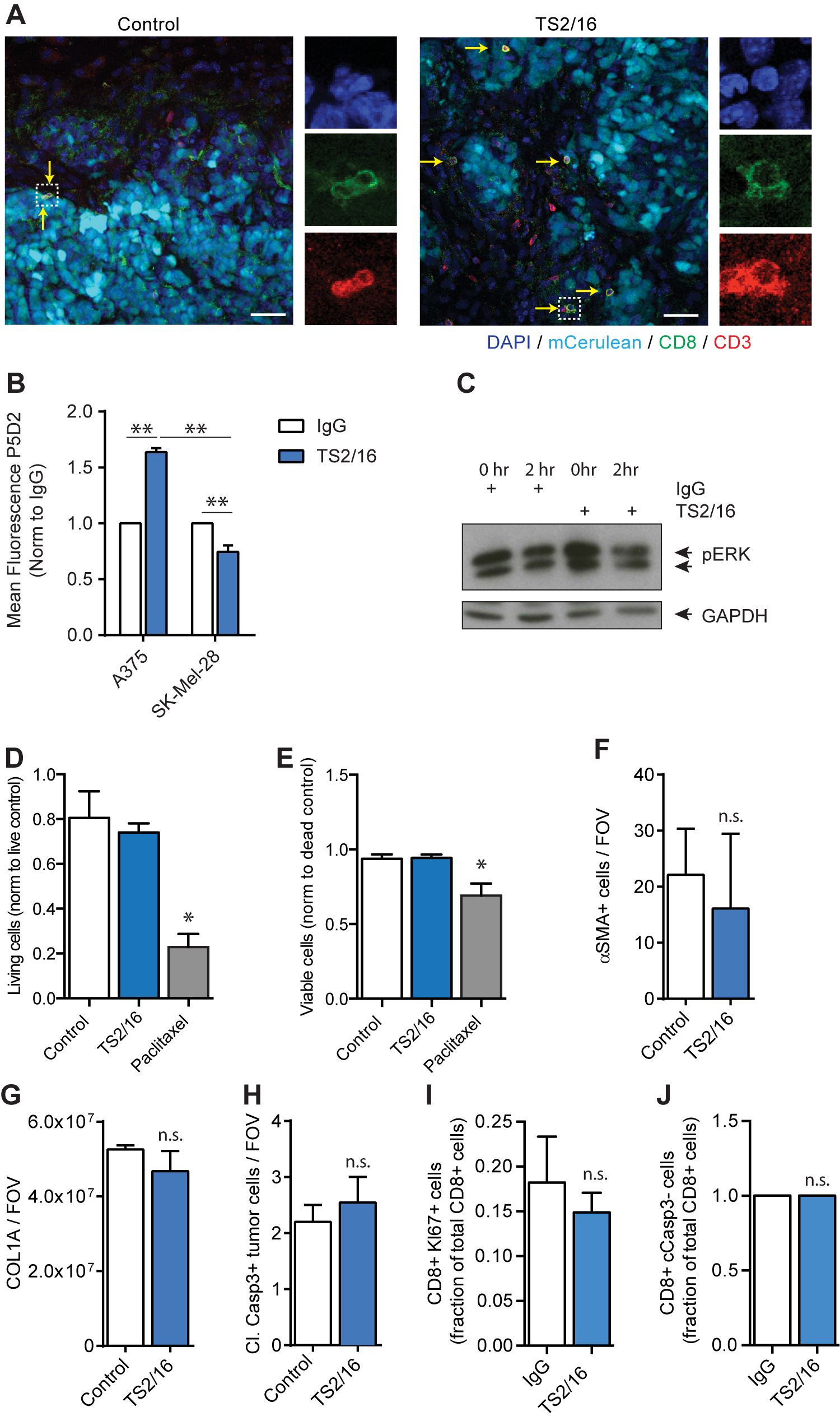

Supplement: S6 Fig — (A) Micrographs of IgG or TS2/16 treated tumors stained for DAPI, CD8 and CD3. CD3+CD8+ T cells are indicated by a yellow arrow. A zoom in of the boxed area is shown on the right. Scale bar, 50 μm. (B) Quantification of FACS data averaging the mean fluorescence intensity for A375 and SK-Mel-28 cells stained with P5D2, measuring total integrin β1. Graphs are normalized to IgG control. N = 3 experiments. (C) Western blot of A375 cells treated with IgG or TS2/16 for 0 or 2 hours stained for pERK and GAPDH. (D) In vitro A375 proliferation assay. Proliferation is measured by analyzing the green fluorescence (Calcein-AM, 530 nm) as a measure for living cells and normalizing it to a live control. N = 3 experiments performed in triplicate. (E) In vitro A375 cell viability assay. Viability is measured by analyzing red fluorescence (Ethidium homodimer-1, 645 nm) as a measure for dead cells and normalizing it to a dead control. N = 3 experiments performed in triplicate. (F-H) IHC of tumors treated with IgG or TS2/16 for 2 days. The number of αSMA+ cells (F) or Cl. Casp3+ cells (H) per field of view (FOV) was quantified. For Type I collagen the mean fluorescence intensity per FOV for COL1A1 was calculated (G). N ≥ 3 mice per condition. (I-J) IHC of tumors treated with IgG or TS2/16 for 3 weeks. The number of CD8+ KI67+ or CD8+Cleaved Caspase 3+ cells was calculated as a fraction of total CD8+ cells per field of view. N ≥ 3 tumors per condition. Error bar, SEM; Two-way ANOVA with Bonferroni posthoc test (B), one-way ANOVA with Bonferroni posthoc test (D,E), double-sided T-test (F-J). * P-value ≤ 0.05, n.s. P-value > 0.05. (TIF) [file pone.0175300.s006.tif]
