# Supplementary material for: Integrin β1 activation induces an anti-melanoma host response
Source: PLoS One. 2017 Apr 27;12(4):e0175300. doi: 10.1371/journal.pone.0175300 (PMC5407755; doi:10.1371/journal.pone.0175300)
Supplement: S1 File — This file contains supplementary methods regarding the bioinformatics analyses that were performed. (DOCX) [file pone.0175300.s011.docx]

Integrin β1 / TGF-β1 activation induces an anti-tumor host response in melanoma

**Laila Ritsma^1,2,^** ^#a^**, Ipsita Dey-Guha^1,2,^** ^¶^**, Nilesh Talele^1,2,^** ^¶^**, Xavier Sole^1,2^, Salony^1,2^, Joeeta Chowdhury^1^, Kenneth N. Ross^1,2^, Sridhar Ramaswamy^1,2,3,4,5,^ ***

Supporting Methods

**TCGA RNASeqV2 Expression Correlation and Outcome Analysis.** Human melanoma patient data was obtained from TCGA to evaluate the *in vitro* and *in vivo* models. TCGA melanoma (SKCM) RNASeqV2 expression data and the associated clinical information for those samples (37) that was used for model evaluation was downloaded from the TCGA data matrix access portal (<http://cancergenome.nih.gov/>). TCGA RNASeqV2 data was downloaded during April 2015 and TCGA clinical data was downloaded during December 2015. Follow-up clinical data files were merged with the original clinical data file to ensure that the most up-to-date patient follow-up information was used for survival analysis. Downloaded TCGA data was loaded into and analyzed in R. The 469 TCGA SKCM samples that had both clinical information and RNASeqV2 data were used in correlation analysis, Gene Set Enrichment Analysis (GSEA), and survival analysis. S1 Table lists the names of those 469 TCGA SKCM samples and their TGFB1 expression and updated clinical information. Of these 469 TCGA SKCM samples, the ‘submitted_tumor_site’ of was listed as being ‘Regional Lymph Node’ for 222 samples, Distant Metastasis’ for 68 samples, ‘Primary Tumor’ for 102 samples, ‘Regional Cutaneous or Subcutaneous Tissue (includes satellite and in-transit metastasis)’ for 74 samples, ‘[Not Available]’ for 2 samples, and ‘[Discrepancy]’ for 1 sample.

The heat map image of Fig 2A was created using data from the 469 SKCM samples with the samples ordered from lowest TGFB1 expression on the left to highest TGFB1 expression on the right. The top panel of the heat map shows the row normalized log2 RNASeqV2 RSEM expression of TGFB1 where dark blue represents the lowest expression and red represents the highest expression. The middle panel shows an Immune Score (‘Imm Score’) row and a Non-Synonymous Mutation rate row (‘Non-syn Mut’). The Immune Score shows the ‘LYMPHOCYTE.SCORE’ value from Table S1D of the 2015 Cell paper on TCGA SKCM (37) where the ‘LYMPHOCYTE.SCORE’ (0-6) is the sum of the ‘LYMPHOCYTE.DENSITY’ (0-3) and ‘LYMPHOCYTE.DISTRIBUTION’ (0-3) scores from a panel of pathologists. Samples that were not scored have a grey box. The Non-Synonymous Mutation rate shows the row normalized non-synonymous mutation rate (‘rate_non’) from GISTIC 2.0 processed copy number array data analysis downloaded from firebrowse.org (downloaded in April 2015). On the right side of the panel, Pearson correlation to log2 TGFB1 (for Immune Score) and significance (* p<0.05 and ** p<0.001) from t-test for tumor site vs. other or correlation test is shown. The bottom panel of Fig 2A shows the log2 RNASeqV2 expression for the 29 genes of the tumor microenvironment signature along with additional rows for log2 SERPINE1 (PAI-1) expression, which is a measure of TGFB1 protein activation, and a meta-gene for beta-integrin signaling. The beta-integrin ITGB1 meta-gene was derived from the 6 validated genes in the beta-integrin signaling pathway (VAV2, EPB41L1, CORO1A, MTOR, CCT4, and GJB3) (37). Each sample’s ITGB1 meta-gene value is found by taking the mean of the log2 RNASeqV2 values for all 6 genes in the signature. The 29 genes of the tumor microenvironment signature are listed in S2 Table. The tumor microenvironment signature is formed out of genes that indicate the presence of T-Cells, Cytolytic activity (CYT), Checkpoint proteins, cancer associated fibroblasts (CAF), Vessels, M1 or M2 macrophages (M1 or M2 MP), and cytokines. The gene symbol along with the gene category color bar is shown to the left of the heat map. On the right of the panel, Pearson correlation to log2 TGFB1 and significance (* p<0.05 and ** p<0.001) from correlation test (cor.test() in R) is shown. S5B Fig was constructed similarly to Fig 2A except all samples whose ‘submitted_tumor_site’ that was listed as ‘Regional Lymph Node’ were left out to show that the patterns and correlations of S5B Fig hold even without the lymph node samples.

Gene Set Enrichment Analysis (GSEA) was used to evaluate the association of TGFB1 with the T Cell and Stroma cell tumor microenvironment sub-signatures as shown in Fig 2B and D (28,29). GSEA was run in the 469 TCGA SKCM RNASeqV2 samples using Pearson correlation with TGFB1 to rank genes and 2500 permutations of the TGFB1 expression phenotype to obtain p-values. The T Cell Immune Gene Signature contained 8 genes that were considered good markers for T Cells as listed in S2 Table. The Stroma Related Gene Signature contains 25 genes that were considered good markers for stroma cells and are listed in S2 Table. This GSEA run was repeated for S5A Fig except for S5A Fig all 222 samples whose ‘submitted_tumor_site’ that was listed as ‘Regional Lymph Node’ were left out.

The Kaplan-Meier plot and the log-rank p-value for TCGA SKCM of Fig 3C was generated using R with the 133 of 469 TCGA SKCM samples from patients that had RNASeqV2 data from either distant metastases (60) or regional cutaneous or subcutaneous tissue (which includes satellite and in-transit metastasis) (73) and clinical information with at least 6 months of follow-up information or a death event. The Kaplan-Meier plot and the log-rank p-value for TCGA SKCM of S4B Fig was generated using R with the 444 of 469 TCGA SKCM samples from patients that had RNASeqV2 data and clinical information with at least 6 months of follow-up information or a death event. The Kaplan-Meier plot used overall survival with death from any cause as the end point and patients still alive at their last follow-up were censored at their last follow-up time. The 444 TCGA SKCM samples were split according whether TGFB1 expression measured by RNASeqV2 fell above or below median expression (10.21). The Kaplan-Meier plot of S4D Fig was produced in a similar fashion except that the samples were further limited to the 278 samples with RNASeqV2 data, MutSig v2.0 processed mutation data, and at least 6 months of follow-up information or a death event and the TGFB1 high and low groups were further split according to the level of non-synonymous mutation rates. The mutation splits for the samples were based upon whether non-synonymous mutation rates (from MutSig v2.0 processed mutation data downloaded from firebrowse.org in April 2015) were high or low (where high indicates ≥ P=0.2 quantile (3.55e-06) and low indicates < P=0.2 quantile).

RNASeqV2 and clinical data was also downloaded for 32 other cancer types that were available from the TCGA data matrix access portal (<http://cancergenome.nih.gov/>). The cancer data types and number of samples are listed in S4 Table. As with TCGA SKCM, follow-up clinical data files were merged with the original clinical data file to ensure that the most up-to-date patient follow-up information was used for survival analysis. Each TCGA data set was analyzed with a method similar to the TCGA SKCM data with overall survival analyzed using a TGFB1 median split of samples from patients with at least 6 months of follow-up information or a death event, GSEA run using Pearson correlation with TGFB1 for the TS2/16 related gene sets, Pearson correlation between RNASeqV2 TGFB1 expression and SERPINE1 expression to determine whether TGFB1 is activated, and Pearson correlation between RNASeqV2 TGFB1 expression and the Astier beta-integrin meta-gene to determine whether ITGB1 is active. The results of all these analyses in the TCGA data sets are shown in S4 Table. The TCGA data set summary heat map plot of Fig 3 summarizes the analysis of the collected TCGA data sets with a heat map with rows for ITGB1 activity as measured by the 6 validated genes in the Astier beta-integrin signaling pathway, TGFB1 activity as measured by SERPINE1 (PAI-1) expression, the TS2/16 Immune Gene Signatures for enrichment (both the T-Cell and stroma related sub-signatures), mutation rates from MutSig v2.0 analysis of mutation data, and overall survival based upon TGFB1 expression levels. Within the heat map of Fig 3, only TCGA datasets with values with p<0.05 are shown (values that are not significant are replaced with a black block).

**TCGA SKCM Clinical Correlate Analysis.** Analysis of clinical and pathological correlates with TGFB1 expression was performed using the curated clinical and pathological data for the TCGA SKCM data contained in the S3 Table: Patient Centric Data from the Cell 2015 paper (37). Each of the clinical or pathological parameters was tested for association with TGFB1 expression as measured by the RNASeqV2 data using a t-test for binary clinical or pathological parameters (such as cancer gene mutation status) or a Pearson correlation test for multi-level or continuous clinical or pathological parameters (such as RPPA protein expression).
